# Alkaloid binding to opium poppy major latex proteins triggers structural modification and functional aggregation

**DOI:** 10.1038/s41467-022-34313-6

**Published:** 2022-11-09

**Authors:** Natali Ozber, Samuel C. Carr, Jeremy S. Morris, Siyu Liang, Jacinta L. Watkins, Kristian M. Caldo, Jillian M. Hagel, Kenneth K. S. Ng, Peter J. Facchini

**Affiliations:** 1 Department of Biological Sciences, University of Calgary, Calgary, Alberta T2N 1N4 Canada; 2 Present Address: Department of Biology, Washington University, St. Louis, MO 63130-4899 USA; 3 Present Address: Department of Chemistry and Biochemistry, University of Windsor, Windsor, Ontario N9B 3P4 Canada

**Keywords:** Secondary metabolism, X-ray crystallography, Plant cell biology

## Abstract

Opium poppy accumulates copious amounts of several benzylisoquinoline alkaloids including morphine, noscapine, and papaverine, in the specialized cytoplasm of laticifers, which compose an internal secretory system associated with phloem throughout the plant. The contiguous latex includes an abundance of related proteins belonging to the pathogenesis-related (PR)10 family known collectively as major latex proteins (MLPs) and representing at least 35% of the total cellular protein content. Two latex MLP/PR10 proteins, thebaine synthase and neopione isomerase, have recently been shown to catalyze late steps in morphine biosynthesis previously assigned as spontaneous reactions. Using a combination of sucrose density-gradient fractionation-coupled proteomics, differential scanning fluorimetry, isothermal titration calorimetry, and X-ray crystallography, we show that the major latex proteins are a family of alkaloid-binding proteins that display altered conformation in the presence of certain ligands. Addition of MLP/PR10 proteins to yeast strains engineered with morphine biosynthetic genes from the plant significantly enhanced the conversion of salutaridine to morphinan alkaloids.

## Abstract (web summary)

Opium poppy accumulates alkaloids in the specialized cytoplasm of laticifers known as latex. Here the authors show that the binding of alkaloids to the abundant major latex proteins triggers quaternary structural modification that mediates a functional role for protein aggregation.

## Introduction

Plants have evolved diverse strategies to store specialized metabolites, often in large quantities. These compounds can be hydrophobic (e.g., volatiles, oils, resins) or polar (e.g., flavonoids, alkaloids, glycosides), chemically inert or reactive, and frequently cytotoxic^1^. Specialized metabolite accumulation generally occurs in specialized cellular, extracellular, or subcellular compartments, including trichome cavities^2,3^, vacuoles^4^, plastids^5^, S-cells^6^, cuticle tissues^7^, nectaries^8^, and laticifers^9^. Opium poppy (*Papaver somniferum*) is a member of the Papaveraceae, one of several families within the Ranunculales order known for the substantial accumulation of benzylisoquinoline alkaloids (BIAs). Although the opium poppy is thought to originate from the Mediterranean region, its domestication dates back to the Neolithic era has resulted in global cultivation for both licit and illicit agriculture^10^. Opium poppy accumulates BIAs in a phloem-associated network of laticifers with morphine, codeine, and thebaine, along with the non-opiates papaverine and noscapine, as the most abundant alkaloids.

Opium poppy employs at least 14 enzymes localized in two different cell types, sieve elements of the phloem and laticifers (Supplementary Figs. 1, 2), to produce opiate alkaloids from tyrosine-derived precursors. Apoplastic translocation of BIA products or intermediates between sieve elements and laticifers involves plasma membrane transporters, including a family of uptake permeases^11^. The ultimate accumulation of numerous BIAs in the cytoplasm (i.e., the latex) of laticifers often exceeds individual aqueous solubility limits (e.g., 700 mM or higher)^12^. Numerous detailed studies on the intracellular anatomy of opium poppy laticifers were unable to address how or where hydrophobic BIAs were stored within these highly specialized cells. Various theories have been purported, including early suggestions that accumulation occurs in or at the periphery of large, membrane-bound vesicles derived from dilating ER^13–15^. Subdivision of the central vacuole during maturation was proposed as the cellular origin of these vesicles^16^. Notably, laticifers feature a soluble proteome dominated (>35%) by a single family of proteins with no clear function^17,18^. Members of the pathogenesis-related (PR)10 family, also known as Bet v 1 or major latex proteins (MLPs), belong to the wider ‘StARkin’ lipid-transfer protein (LTP) classification with diverse physiological roles in plants, including sterol trafficking, lipid ‘sensor’ activity, and modulation of transcription factor function^19^. Bet v 1 proteins descended from an ancient common ancestor that diversified into numerous subfamilies displaying relatively low sequence identity but retaining a conserved structural fold that imparts the ability to bind hydrophobic ligands^20^. The expansion of protein databases containing structural information has facilitated comparisons that have revealed similarities in tertiary structure among proteins exhibiting limited amino acid sequence similarity. The striking structural similarity of Pru av 1 (a Bet v 1 protein from cherry, *Prunus avium*) to the lipid-binding domain of human cholesterol-binding protein MLN64 revealed the existence of a large protein superfamily with a common fold^21^. This superfamily is denoted as the Bet v 1-like clan in the Pfam protein family database [PfamC:CL0209] and the Bet v 1-like superfamily in the Structural Classification of Proteins (SCOP) database [SCOP:d.129.3].

In opium poppy, the purported ability of Bet v 1 proteins to bind hydrophobic alkaloids potentially underpinned their recruitment as biosynthetic enzymes. In morphine biosynthesis, at least three enzymes belong to the MLP/PR10 protein family: norcoclaurine synthase (NCS)^22^, thebaine synthase (THS)^23^, and neopinone isomerase (NISO)^24^ (Supplementary Fig. 1). Evolution of catalytic proteins from ligand-binding, non-catalytic ancestors is an emerging paradigm^25,26^ encouraging further investigation of how structural conformation and ligand binding evolves as proteins acquire catalytic capabilities^27^. Furthermore, it is possible that presumed non-catalytic MLP/PR10 proteins support BIA metabolism in an auxiliary role. In plants, auxiliary proteins play key roles as modifiers of metabolic flux, production enhancers, stereoselectivity mediators, intermediate transporters, and regulators^28^. Examples include non-catalytic chalcone isomerase-like proteins (CHILs) that support demethylxanthohumol biosynthesis in hop (*Humulus lupus*)^29^ and the Bet v 1 protein Fra a, shown to control flavonoid pigment metabolism in strawberry (*Fragaria* x *ananassa*)^30^. Both catalytic and non-catalytic roles have been assigned to single protein types or even individual proteins. For example, certain plant glutathione *S*-transferases (GSTs) play a role in ligand binding and/or transport, as also in *S*-glutathionylation^31^.

In this paper, we use sucrose density-gradient fractionation coupled with shotgun proteomics, differential scanning fluorimetry, isothermal calorimetry, and X-ray crystallography, to show that MLP/PR10 proteins bind to major BIAs found in opium poppy latex. We demonstrate a physiological role for both catalytic and noncatalytic MLP/PR10 proteins in BIA metabolism in yeast engineered with morphine pathway biosynthetic genes and using virus-induced gene silencing (VIGS) in opium poppy. Our study is linked to longstanding observations of ‘dense vesicles’ in opium poppy latex and suggests that alkaloid binding to MLP/PR10 proteins initiates the formation of biomolecular condensates involved in the sequestration of BIAs.

## Results

### MLP/PR10 proteins co-sediment with major BIAs in opium poppy latex

**Fig. 1:**
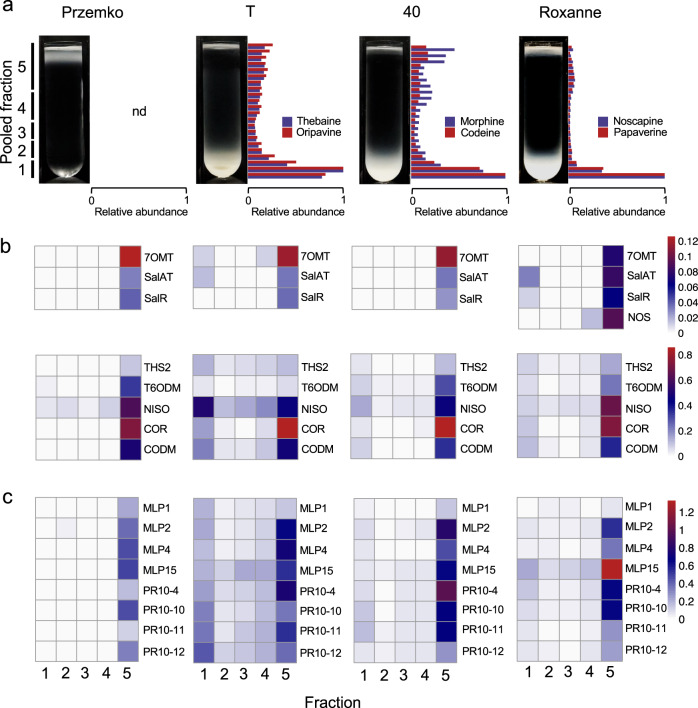
Sucrose-gradient analysis of opium poppy latex. **a** Images of sucrose density gradients run with Przemko, T, 40 or Roxanne latex, and the relative abundance of selected BIAs in gradient fractions (blue and red bars). The pooled fractions used for shotgun proteomics are indicated on the left side. Alkaloid levels were normalized to the fraction with the greatest abundance. **b** Heat maps showing the relative abundance of BIA biosynthetic enzymes in proteomes of pooled fractions. **c** Heat maps showing the relative abundance of eight major MLP/PR10 proteins in pooled fractions. Color scales indicating percentage of the total spectral counts in each proteome are shown on the right side of panels **b**, **c**. Abbreviation: nd, not detected. Note that pooled fractions 1–5 are comprised of unpooled fractions 1–22 (Supplementary Figs. 4 and 6). Enzyme abbreviations and role in alkaloid biosynthetic pathway(s) are detailed in Supplementary Figs. 1, 2.

Fresh latex from opium poppy Przemko, T, 40, and Roxanne cultivars was fractionated on a 14–66% sucrose density gradient. Based on BIA content, these cultivars represent four distinct chemotypes: (i) alkaloid-free ‘Przemko’, (ii) high thebaine, but morphine/codeine-free ‘T’, (iii) high opiate (i.e. morphine, codeine, and thebaine) ‘40’, and (iv) high opiate and noscapine/papaverine-containing ‘Roxanne’^12,32^. Gradient profiles for each chemotype displayed a distinct pattern of high-density protein bands (Fig. 1a). The BIA profile of gradient fractions showed that major alkaloids (i.e., morphine, codeine, thebaine, oripavine, noscapine, and papaverine) mostly migrated to the highest density fraction (~1.3 g mL^−1^) and, to a much lesser extent, low-density fractions (Fig. 1a; Supplementary Figs. 3, 4). The composition and relative abundance of specific proteins in gradient fractions were determined using comparative shotgun proteomics (Supplementary Data 1). Latex gradient-fraction proteomes were initially searched for established organellar markers, and their distribution in each pooled fraction was determined (Supplementary Table 1). All eight morphine pathway enzymes [i.e., salutaridinol reductase (SalR), salutaridinol 7-*O*-acetyltransferase (SalAT), THS, thebaine 6-*O*-demethylase (T6ODM), NISO, codeinone reductase (COR), and codeine *O*-demethylase (CODM)] and reticuline 7-*O*-methyltransferase (7OMT) were detected in latex proteomes (Fig. 1b). NOS, a noscapine biosynthetic enzyme, was found only in the Roxanne proteome (Fig. 1b; Supplementary Fig. 2b). In Przemko, morphine pathway enzymes were primarily found in the low-density fraction. In contrast, in alkaloid-accumulating chemotypes (Roxanne, 40, and T), BIA biosynthetic enzymes were mostly found in two gradient fractions: the highest-density fraction (~1.3 g mL^−1^) and the lowest-density fraction (Fig. 1b). Along with BIA biosynthetic enzymes, eight MLP/PR10 family proteins (i.e., MLP1, MLP2, MLP4, MLP15, PR10-4, PR10-10, PR10-11, and PR10-12) were abundant in all chemotypes. However, the gradient distribution pattern of these MLP/PR10 proteins varied among chemotypes (Fig. 1c; Supplementary Table 2). For example, a greater proportion of MLP/PR10 proteins sedimented in T compared with 40 and Roxanne (Fig. 1c). Of all cultivars, T exhibits the highest absolute total abundance of BIAs; in comparison, 40 and Roxanne contain only approximately 80 and 70%, respectively, of the alkaloid content (i.e., per milligram dry latex) found in T^32^. Furthermore, proteomics results indicate 40 and Roxanne contain only 80 and 83%, respectively, of the relative PR10 content in T (Supplementary Table 2). In Przemko, major MLP/PR10 proteins were almost exclusively detected in the lowest-density fraction. In alkaloid-accumulating chemotypes, MLP/PR10 proteins sedimented to both the lowest- and highest-density fractions. Other minor MLP/PR10 proteins (Supplementary Data 1) displayed fractionation patterns consistent with those of major MLP/PR10 proteins (Supplementary Fig. 5a).

**Fig. 2:**
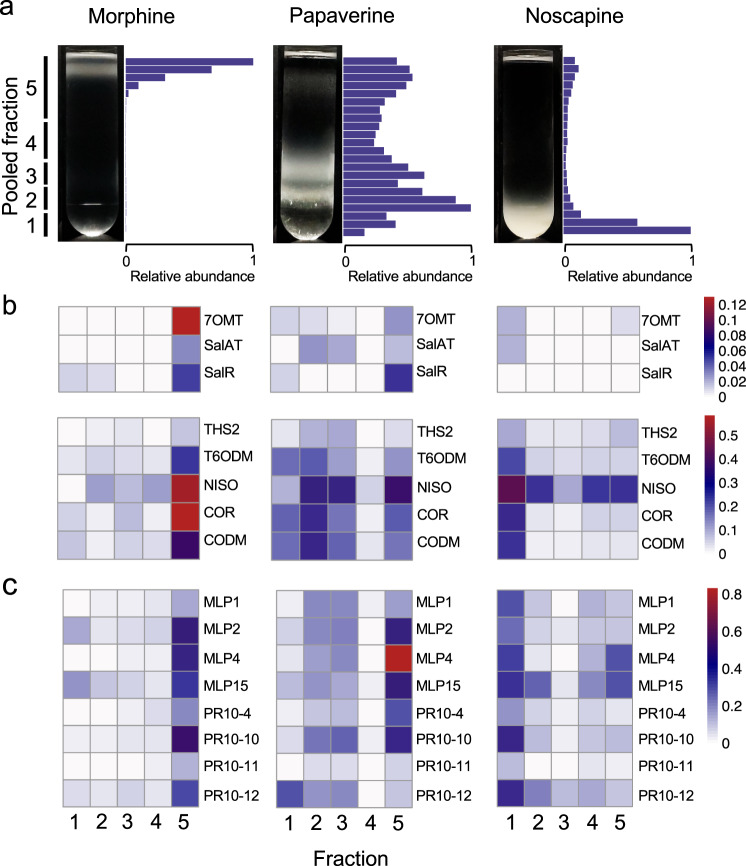
Sucrose-gradient analysis of alkaloid-added Przemko latex. **a** Images of sucrose density gradients run with Przemko latex containing exogenous morphine, papaverine or noscapine, and the relative abundance of the exogenous alkaloid in gradient fractions (blue bars). The pooled fractions used for shotgun proteomics are indicated on the left side. Alkaloid levels were normalized to the fraction with the greatest abundance. **b** Heat maps showing the relative abundance of BIA biosynthetic enzymes in proteomes of pooled fractions. **c** Heat maps showing the relative abundance of eight major MLP/PR10 proteins in pooled fractions. Color scales indicating percentage of the total spectral counts in each proteome are shown on the right side of panels **b**, **c**. Abbreviation: nd, not detected. Note that pooled fractions 1–5 are comprised of unpooled fractions 1–22 (Supplementary Figs. 4 and 6). Enzyme abbreviations and role in alkaloid biosynthetic pathway(s) are provided in Supplementary Figs. 1 and 2.

The co-sedimentation of BIA biosynthetic enzymes and MLP/PR10 proteins was further investigated by the independent addition of exogenous noscapine, papaverine, or morphine to the nearly alkaloid-free latex of the Przemko chemotype, followed by sucrose density-gradient fractionation (Fig. 2). This exogenous addition of BIAs to latex post-extraction represented a direct, albeit artificial approach, compared with the fractionation of whole latex and was performed only using the Przemko cultivar; thus, results are shown separately to improve clarity. The addition of papaverine or noscapine to Przemko latex resulted in one or more visibly insoluble dense aggregate bands. In contrast, this higher-density material was not observed in Przemko latex containing exogenous morphine (Fig. 2a). Shotgun proteomics analysis showed that the distribution of alkaloid biosynthetic enzymes corresponded with the relative abundance of various alkaloids in all three gradients (Fig. 2a, b). For example, the relatively low level of morphine in pooled fraction 5 (Fig. 2a) coincided with a predominance of enzymes in the same pooled fraction (Fig. 2b). In contrast, the broader distribution of papaverine throughout the pooled fractions, with a small peak in fraction 2, correlated with comparatively diffuse distributions of many enzymes, many of which (i.e., 7OMT, SalAT, THS2, NISO, COR, CODM) showed peak abundance in the same fraction. Noscapine was detected mostly in pooled fraction 1, which correlated with a maximum abundance of biosynthetic enzymes in this fraction. Biosynthetic enzymes were primarily detected in low-density Przemko latex fractions containing exogenous morphine, consistent with the abundance of morphine in these fractions. The inclusion of papaverine or noscapine in Przemko latex led to the enrichment of almost all detected alkaloid biosynthetic enzymes in higher-density fractions. In all three gradients, the fractionation patterns of MLP/PR10 proteins largely corresponded with those of detected biosynthetic enzymes (Fig. 2c; Supplementary Fig. 5b). As the amount of exogenous BIAs added to Przemko latex was greater than that naturally occurring quantities (see Methods), and MLP/PR10 levels show some variances with respect BIA-containing cultivars, direct comparisons with the fractionated latex of T, 40 or Roxanne were not made. However, an informative comparison was made with respect to control experiments, which showed that morphine was abundant in lower-density fractions but absent in higher-density fractions (Supplementary Fig. 6a), while papaverine and noscapine were distributed along the gradient with papaverine enriched in lower-density fractions (Supplementary Fig. 6b).

### MLP/PR10 proteins bind to BIAs

**Fig. 3:**
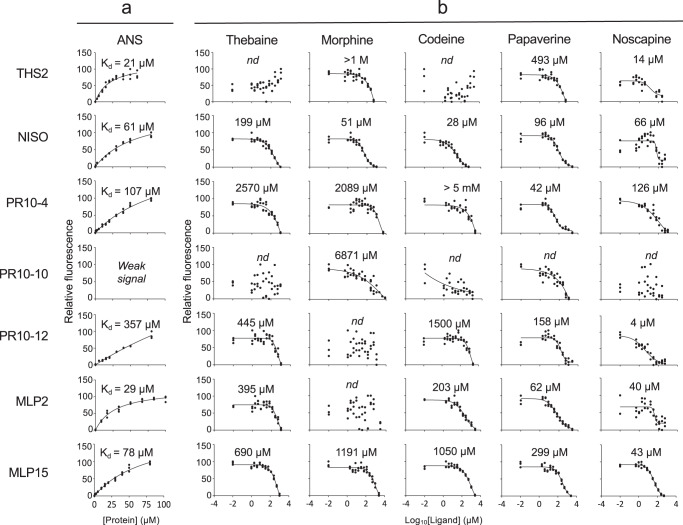
Relative ANS fluorescence, displacement curves and calculated IC_50_ values for the most abundant MLP/PR10 proteins and the major alkaloids in opium poppy latex. **a** Affinity between ANS and each MLP/PR10 protein was determined directly based on fluorescence and the corresponding *K*_d_ value was calculated. ANS concentration (10 µM) was fixed and MLP/PR10 protein concentration was varied from 0 to 100 µM. A one-site hyperbolic model was fit to determine *K*_d_ values. **b** BIA binding-induced decrease in fluorescence caused by ANS displacement and corresponding IC_50_ values. Assuming competitive inhibition, dose-response binding curves were fit, and apparent *K*_d_ were calculated from the ANS affinity, and IC_50_ values were determined (Supplementary Table 3). Data shown is representative of three fully independent experiments. *Weak signal* indicates insufficient ANS fluorescence to determine *K*_d_. Abbreviation: nd, not determined.

**Fig. 4:**
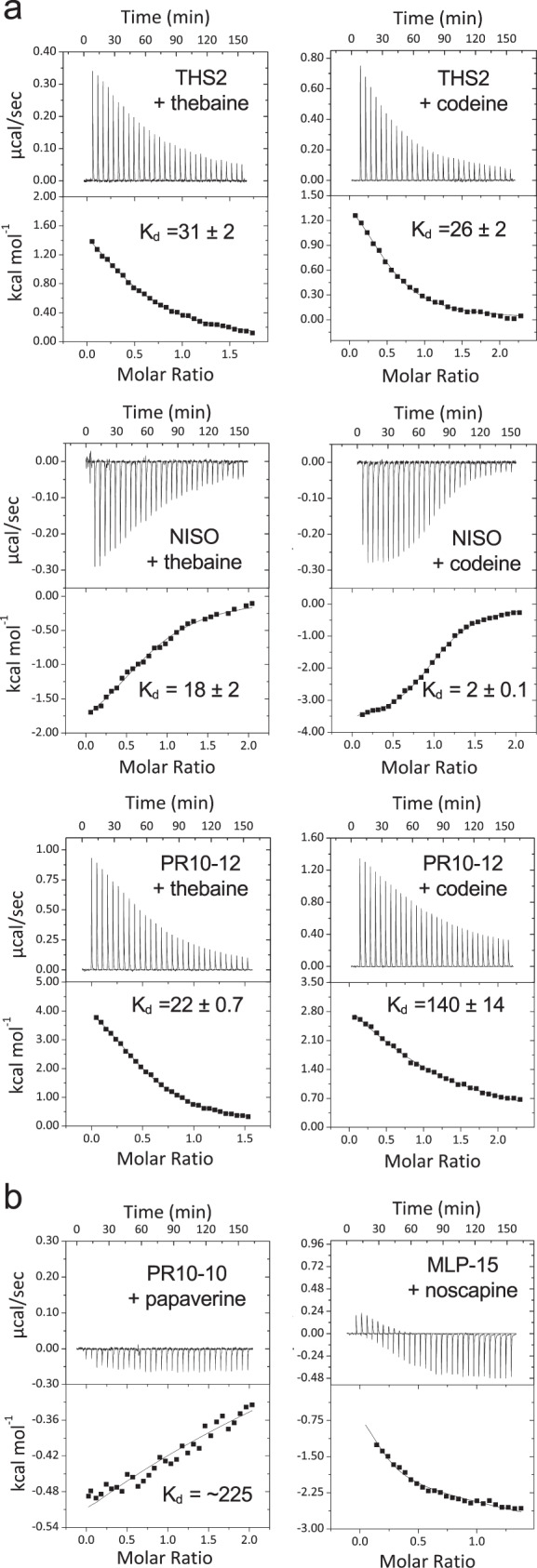
Isothermal titration calorimetry analysis of THS2, PR10-12, NISO, PR10-10 and MLP15. **a** ITC thermograms for thebaine and codeine binding to THS2, PR10-12, and NISO. *K*_d_ values represent mean ± standard error. Each panel set is representative of three fully independent experiments. All experiments were performed in PBS buffer, pH 7.4. **b** ITC thermograms for noscapine binding to MLP15 and papaverine binding to PR10-10. All experiments were performed in CBS buffer. Each panel set is representative of three fully independent experiments.

Ligand-binding properties of the major MLP/PR10 proteins (i.e., THS2, NISO, PR10-4, PR10-10, PR10-12, MLP2, and MLP15; Supplementary Fig. 7) were analyzed using 8-anilinonaphthalene-1-sulfonic acid (ANS) displacement assays (Fig. 3; Supplementary Table 3) and isothermal titration calorimetry (ITC) (Fig. 4; Supplementary Table 4). Ligands included the major latex alkaloids thebaine, morphine, codeine, papaverine, and noscapine. Although PR10-10 did not bind ANS in a typical manner (Fig. 3a), precluding IC_50_ determination, binding curves were determined for morphine and papaverine. Noscapine and papaverine showed the strongest binding affinity to all tested MLP/PR10 proteins in ANS-displacement assays (Fig. 3) with IC_50_ values from 4 to 126 μM and 42 to 493 μM, respectively. PR10-10 also exhibited atypical binding with noscapine. ITC supported the binding of MLP15 to noscapine, and of PR10-10 to papaverine. Both results were associated with atypical thermodynamic modulations that occurred during the injection of either blank or protein-containing samples (Fig. 4; Supplementary Table 4). Morphine exhibited binding to all tested MLP/PR10 proteins, with the exception of PR10-12 and MLP2, yielding IC_50_ values from 51 μM to >1 M. Codeine and thebaine demonstrated binding to all tested MLP/PR10 proteins, excluding THS and PR10-10, with IC_50_ values from 28 μM to >5 mM and 199 μM to 2.6 mM, respectively. Using ITC, THS2 was confirmed to bind thebaine, which is an enzymatic product of the protein, with an apparent *K*_d_ of 31 μM (Fig. 4). ITC further confirmed the binding of thebaine to PR10-12 (*K*_d_ 22 μM) and NISO (*K*_d_ 18 μM) and the binding of codeine to THS2 (*K*_d_ 26 μM), PR10-12 (*K*_d_ 140 μM), and NISO (*K*_d_ 2 μM). Excluding the binding of codeine to PR10-12, ITC N-values of 0.5 suggested that a single MLP/PR10 protein binding site could be occupied by two alkaloid ligand molecules (Supplementary Table 4). The binding of either thebaine or codeine to both PR10-12 and THS was endothermic (ΔH + 1868 to +6263 cal/mol) and was, thus, entropically driven (ΔS + 27.2 to +39.3 cal/mol/˚K) resulting in a negative Gibbs free energy change. In contrast, NISO displayed an exothermic binding with a high affinity for thebaine (*K*_d_ 18 μM) and codeine (2 μM). THS2 and PR10-12 were predicted to exhibit a high affinity for noscapine from ANS-displacement experiments, but reliable binding constants were not obtained using ITC. In such cases, binding was associated with low enthalpy change (<700 cal/mol), yielding high signal-to-noise ratios (Supplementary Table 4).

### Crystal structure of PR10-10

**Fig. 5:**
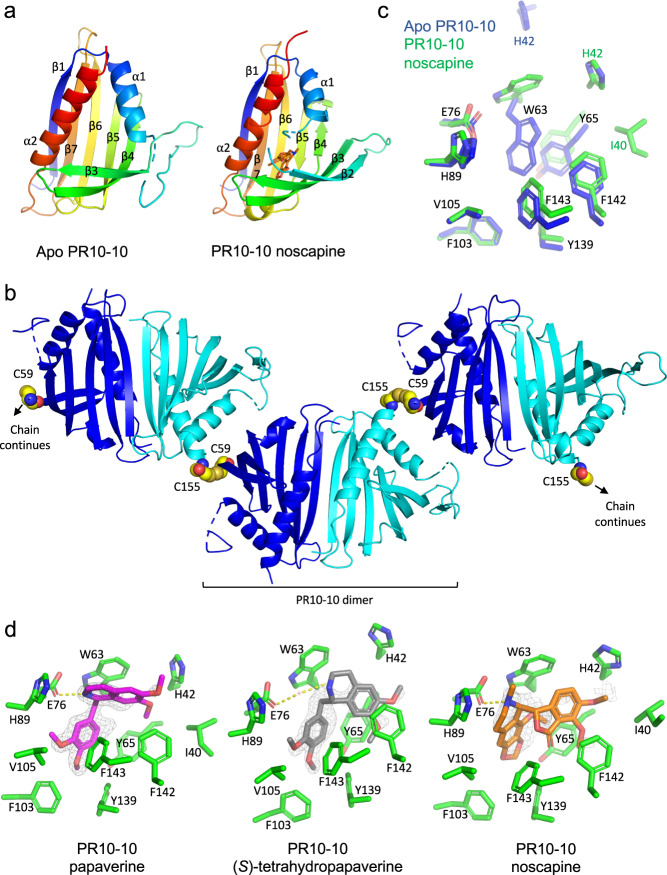
Crystal structure Apo-PR10-10 and PR10-10 in complex with noscapine, papaverine, and (*S*)-tetrahydropapaverine. **a** Front view of single PR10-10 protomers for the *apo* and noscapine complexed structures of PR10-10. The rainbow coloring scheme starts at the C-terminus in red to the N-terminus in blue. Helices and β strands are numbered accordingly. Noscapine is shown in orange. **b** *Apo* PR10-10 ‘in-crystal’ polymer and PR10-10 dimer. Protomers from the PR10-10 dimer are shown in cyan and blue. Disulfides are shown as yellow spheres. **c** comparison of the *apo* and noscapine complexed PR10-10 binding pocket. Dark blue atoms and bonds correspond to *apo* PR10-10 carbons, green atoms and bonds to papaverine complexed PR10-10 carbons, light blue to nitrogen atoms, red to oxygen, and yellow to sulfur. Noscapine is not shown. **d** PR10-10 binding pocket with complexed papaverine in magenta, (*S*)-tetrahydropapaverine in grey, and noscapine in orange. Simulated annealing |Fo | - |Fc| omit map at 3σ is represented as grey mesh. Start and end temperature for simulated annealing were 5000 K and 300 K. Green atoms and bonds correspond to PR10-10 carbons, blue to nitrogen atoms, red to oxygen, and yellow to sulfur. Water molecules are shown as red dots. Yellow doted lines correspond to possible hydrogen bonds.

High-quality crystal formation was pursued for MLP/PR10 proteins that fulfilled the following criteria were: (1) overall high abundance in opium poppy latex as indicated by proteomics data and (2) occurrence of the protein in high-density fractions of sedimented latex. Proteins yielding anomalous binding data determined using ANS displacement assays or ITC experiments were not precluded since these experiments were not conclusive for proteins forming insoluble aggregates upon ligand binding. Of all the proteins meeting these criteria, PR10-10 readily formed high-quality crystals with bound alkaloid. The crystal structure of PR10-10 was solved by molecular replacement in the *apo* and *holo* forms with papaverine, (*S*)-tetrahydropapaverine, and noscapine. Data collection and refinement statistics are provided in Supplementary Table 5. The overall structure of PR10-10 reveals a typical Bet v 1-fold. Seven antiparallel β-strands cradle the C-terminal α-helix with the alkaloid binding pocket located in a hydrophobic pocket between the central C-terminal α-helix and the β-sheet (Fig. 5a). Alkaloid binding leads to the ordering of β2 completing the Bet v 1-fold. This isolates the bound alkaloid from the solvent, while the disordering of β2 in *Apo* PR10-10 exposes the binding pocket. *Apo* and *holo* PR10-10 reveal the same homodimer, conserved in THS2, formed by chains A and B in *apo* PR10-10 and through crystallographic symmetry for *holo* PR10-10, forming along the β1 strand of each protomer with two-fold symmetry relating the binding pockets on opposite sides of the dimer interface (Fig. 5b, Supplementary Fig. 8). The crystal packing of *apo* PR10-10 suggests the formation of indefinitely repeating polymeric chains of homodimers. Disulfide linkages between the well-conserved residues C155 from chain A and C59 from chain B link PR10-10 dimers forming ‘in-crystal’ polymers (Fig. 5b). Disulfide-mediated oligomerization is only observed in *apo PR10-10*. Alkaloid binding and subsequent ordering of the β2 strand causes C59 to be buried. Non-reducing SDS-PAGE demonstrates the stability of disulfide linkages outside of the crystal environment (Supplementary Fig. 9). The alkaloid binding pocket of PR10-10 is formed by I40, H42, W63, Y65, E76, H89, F103, V105, Y139, F142, and F143. In the absence of a bound alkaloid, W63 rotates to occupy the hydrophobic binding pocket (Fig. 5c). E76 hydrogen bonds directly with the isoquinoline moiety amine of papaverine and noscapine, and through a water molecule for (*S*)-tetrahydropapaverine (Fig. 5d). The PR10-10 binding pocket adopts the same conformation for the papaverine, (*S*)-tetrahydropapaverine, and noscapine bound complexes. The previously solved structures of THS2^33^ and NCS^34^ demonstrate overall structural conservation with PR10-10, but only THS2 shows strong sequence and binding pocket architecture similarities to PR10-10 (Supplementary Figs. 10 and 11; Supplementary Information). Notably, whereas ITC results suggested that a single MLP/PR10 protein binding-site could be occupied by two alkaloid ligand molecules (Supplementary Table 4), crystallography data showed that PR10 −10 bound only a single ligand. The size and flexibility of the binding pocket do not exclude the possibility that two alkaloids could potentially be accommodated. Based on the structural and chemical diversity of PR10/MLP proteins and their alkaloid ligands, respectively, multiple modes of binding might be expected.

### MLP/PR10 proteins modulate alkaloid accumulation in engineered yeast and plants

**Fig. 6:**
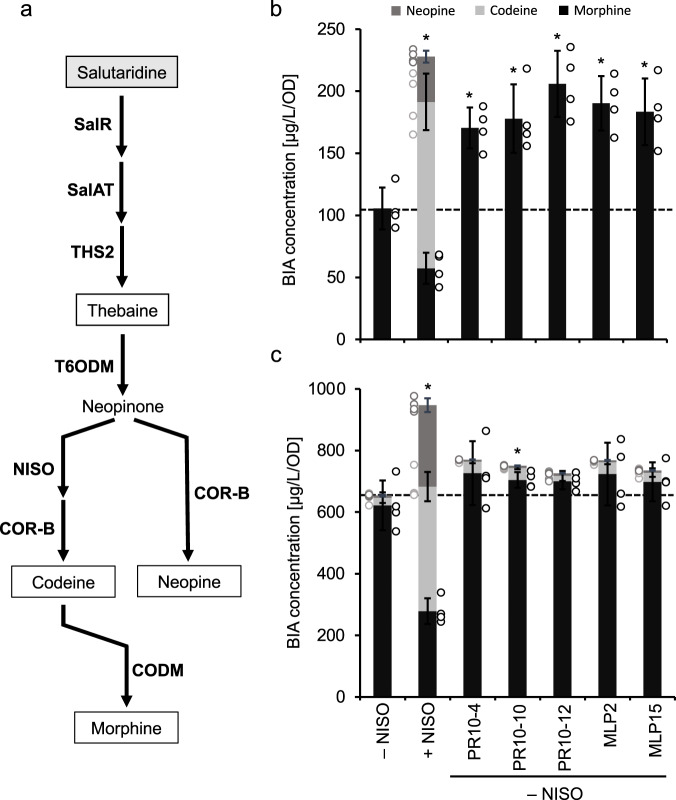
Effect of major MLP/PR10 proteins on morphinan alkaloid accumulation in an opiate-producing yeast strain fed salutaridine. **a** Pathway for the conversion of exogenous salutaridine to codeine, morphine and neopine. Abbreviations: COR-B, codeinone reductase B; CODM, codeine *O*-demethylase; NISO, neopine isomerase; SalAT, salutaridinol 7-*O*-acetyltransferase; SalR, salutaridine reductase; T6ODM, thebaine 6-*O*-demethylase; THS, thebaine synthase. **b** Accumulation of morphine, codeine, and neopine in engineered yeast fed 50 µM exogenous salutaridine for 2 h. **c** Accumulation of morphine, codeine, and neopine in engineered yeast fed 50 µM exogenous salutaridine for 6 h. Bars represent the mean ± standard deviation of 4 biological replicates. Stacked bars show the mean levels of individual morphinan alkaloids ± standard deviation. Asterisks indicate a significant difference (*p* < 0.05; unpaired, two-tailed *t*-test) in alkaloid accumulation.

A physiological role for MLP/PR10 proteins in BIA metabolism was investigated using yeast (*Saccharomyces cerevisiae*) engineered with opium poppy biosynthetic genes encoding enzymes involved in the conversion of salutaridine to morphine. The gene encoding the catalytic MLP/PR10 protein *NISO* was not included in the yeast strain to test the auxiliary, non-catalytic impact of other MLP/PR10 proteins, which resulted in a substantial accumulation of the codeine isomer neopine (a rare metabolite in opium poppy) rather than morphine, the major product in opium poppy plants (Fig. 6). Plasmid-based expression of genes encoding NISO, PR10-4, PR10-10, PR10-12, MLP2, and MLP15 showed the detection of heterologous MLP/PR10 proteins in yeast (Supplementary Fig. 12a). After 2 h incubation with salutaridine, yeast strains expressing individual MLP/PR10 proteins exhibited a significant increase in the accumulation of neopine, or a combination of neopine, codeine, and morphine with the inclusion of NISO, compared with empty-vector controls (Fig. 6b). After 6 h incubation, the effect of MLP/PR10 proteins was reduced, although NISO and PR10-10 continued to show a significant effect on alkaloid accumulation (Fig. 6c). Increased alkaloid titers were also observed in the presence of MLP/PR10 proteins 2 h after feeding thebaine; however, these increases were not significant (*p* < 0.05) (Supplementary Fig. 12b) and were not detected after 6 h (Supplementary Fig. 12c). Virus-induced gene silencing (VIGS) further confirmed a physiological role of MLP/PR10 proteins in BIA metabolism (Supplementary Fig. 13). The pTRV2 construct (pPR10) was designed to target highly conserved regions of the most abundant MLP/PR10 protein genes in opium poppy latex (e.g., *MLP15*, *PR10-10*, and *PR10-4*). Plants infected with *A. tumefaciens* harboring pPR10 showed reduced *NISO*, *PR10-4*, *PR10-12*, *MLP2*, and *MLP15* transcript levels (Supplementary Fig. 13a). Suppression of MLP/PR10 transcript levels was associated with a decrease in the morphine content of the latex, and an increase in the accumulation of noscapine and papaverine (Supplementary Fig. 13b).

## Discussion

Decades of research on opium poppy have culminated in the complete elucidation of complex pathways leading to morphine and noscapine from dopamine and 4-hydroxyphenylacetaldhyde (Supplementary Figs. 1 and 2). In contrast, the physiology of alkaloid storage and a possible role for auxiliary proteins supporting alkaloid biosynthesis and accumulation have not been established. We hypothesized a potential involvement of MLP/PR10 proteins in BIA metabolism, binding, and accumulation in opium poppy based on (1) the reported binding by PR10 proteins and members of the larger Bet v 1-like superfamily to nitrogen-containing hydrophobic metabolites such as aromatic alkaloids^35–37^, (2) the known role of three PR10 proteins (i.e., NCS, THS, and NISO) as BIA biosynthetic enzymes, and (3) the sheer abundance of MLP/PR10 proteins in the latex. We initially sought to determine whether alkaloids, BIA biosynthetic enzymes, and MLP/PR10 proteins were co-localized in the latex using sucrose density-gradient fractionation (Fig. 1). Density-gradient centrifugation is an established technique for the separation of organelles, whereby the sedimentation rate depends on factors including protein content, lipid:protein ratio, and particle size and shape^38,39^. Immediately apparent upon fractionation of BIA-rich opium poppy latex was the partitioning of flocculent material to the top (1.00 – 1.10 g mL^−1^) and ‘bottom’ (>1.24 g mL^−1^) of the gradient (Fig. 1a; Supplementary Fig. 3). Aqueous-insoluble, low-density, lipidic materials were anticipated near the top of the gradient along with cytosolic and/or unfolded proteins exposing interior hydrophobic domains. None of the cellular markers used distinctly co-localized with the dense bottom of the gradient (i.e., fraction 1). Subcellular materials known to sediment to this density (>1.24 g mL^−1^) include insoluble aggregates termed protein bodies, which occur in organisms from bacteria (e.g., inclusion bodies) to mammals (e.g., Russel bodies)^40^. In plants, seed storage proteins form either protein bodies (PBs) derived from rough ER or cisternal-derived protein storage vacuoles (PSVs)^41,42^. Protein bodies are also found in phloem and participate in sieve element occlusion processes^43,44^. Microscopic examination of opium poppy laticifers failed to reveal canonical protein bodies or other subcellular structures with a possible storage function^13,14,45^, although electron-dense regions, or ‘caps’, were shown to form along the inner surfaces of so-called ‘alkaloid vesicles’ in OsO_4_-infiltrated sections. These caps appeared to arise via the condensation of particles observed early in laticifer development^14^ and often featured an ordered stroma described as ‘granular’^46^.

The sedimentation of major latex components to high-density sucrose gradient fractions prompted us to speculate that BIA biosynthetic enzymes and presumed non-catalytic MLPs/PR10 proteins aggregated in complex with alkaloids to form particles with density and size comparable to protein bodies. Such dense aggregates have been noted previously in high-density (i.e., 65–75% sucrose) opium poppy latex fractions, which also showed enrichment in alkaloids^15,47,48^. In studies conducted prior to the availability of shotgun proteomics and affiliated genomics resources, many abundant latex proteins (e.g., THS, NISO, CODM, individual MLP/PR10 proteins, etc.) could not be identified^48^, and quantitative proteomic differences between high- and low-density fractions were difficult to detect. Our current data and previous findings support the occurrence in opium poppy latex of dense aggregates enriched in MLP/PR10 proteins (Supplementary Data 1; Fig. 1c; Supplementary Fig. 5a) and alkaloids (Fig. 1a; Supplementary Fig. 4). Notably, T latex exhibits the highest proportion of MLP/PR10 in ‘dense’ fractions (Fig. 1c). Compared with 40 and Roxanne cultivars, T contains a greater abundance of alkaloids^32^ and a higher relative abundance of MLP/PR10 proteins (Supplementary Table 2), the combination of which could induce more extensive aggregate formation. To further investigate the nature of this proposed protein-alkaloid aggregates, a series of experiments were conducted using the alkaloid-free latex of the opium poppy chemotype Przemko, to which exogenous major latex alkaloids morphine, papaverine, or noscapine were independently added prior to sucrose density-gradient fractionation (Fig. 2). Sucrose density-gradient fractionation of Przemko latex, which is devoid of alkaloids yet contains a full complement of known BIA biosynthetic enzymes and MLP/PR10 proteins, did not display the high-density aggregated material found in gradients run with latex from alkaloid-rich chemotypes (Fig. 1). However, the protein distribution pattern is dramatically altered when individual alkaloids were added to Przemko latex prior to sucrose density-gradient fractionation. Exogenous morphine was more abundant in higher-density fractions than controls in which no latex was added (Supplementary Fig. 6a), coinciding with a similar shift in the sedimentation of BIA biosynthetic enzymes and MLP/PR10 proteins (Fig. 2). Exogenous addition of morphine to Przemko latex did not induce the formation of substantial insoluble protein aggregate and morphine remained largely at the top of the gradient (Fig. 2a). Conversely, endogenous morphine in 40 latex was detected in relatively dense gradient fractions associated with aggregated protein near the bottom of the gradient (Fig. 1a). These apparently incongruent results suggest that (i) morphine has limited capacity to trigger protein aggregate formation compared with more hydrophobic alkaloids (e.g. noscapine and papaverine; Fig. 2a), and (ii) aggregate formation in the plant involves a complex mechanism, perhaps similar to protein body formation at the ER, that is triggered by certain alkaloids, but ultimately provides a ‘compartment’ for the accumulation of all alkaloids, including morphine. Exogenous papaverine and noscapine were substantially more abundant in higher-density fractions, accompanied by the appearance of opaque bands in the gradient and with strong shifts in the sedimentation of BIA biosynthetic enzymes and MLP/PR10 proteins to higher-density fractions (Fig. 2). Future experiments could include (i) testing the effectiveness of different concentrations of various alkaloids to induce protein aggregate formation in Przemko latex or (ii) using purified, recombinant PR10 proteins to deconvolve the role of individual proteins in aggregate formation.

These data suggested that MLP/PR10 proteins undergo tertiary or quaternary structural changes in complex with alkaloids, resulting in the formation of distinct phases or subcellular aggregates responsible for the high levels of BIA accumulation in opium poppy latex. In other plants, specific triggers affect nucleation and ‘swelling’ (polymerization) of insoluble aggregates, such as protein disulfide bond formation^49,50^, shifts in calcium and pH^43^, or unfolded protein response-related signal cascades^51^. MLP/PR10 proteins could form biomolecular condensates, so-called membrane-less organelles, that require specific conditions to separate from the surrounding cellular milieu^52^. Nucleation rates for these condensates can be modulated by biomolecular ‘seeds’ possessing an ability to form weak bonds (e.g., cation−pi interactions; pi-stacking interactions) with other biomolecules. Such interactions overcome forces that keep molecules in the surrounding solution, creating a new ‘phase’ with distinct density, character, and composition.

Unequivocal evidence that latex proteins bind alkaloids is central to the hypothesis that certain alkaloids seed the formation of macromolecular, protein-rich condensates. We showed that all major alkaloids tested bound multiple latex MLP/PR10 proteins with varying affinity (Figs. 3 and 4). As BIA biosynthetic enzymes, THS and NISO are known to bind at least their alkaloid substrates; for example, THS and NISO substrate kinetics have been determined for the alkaloids salutaridinol-7-*O*-acetate (*K*_A_ = 22 μM)^23^ and codeinone (*K*_m_^app^ = 48 μM)^24^, respectively. Indeed, NISO exhibited relatively low IC_50_ values for most tested alkaloids in ANS displacement assays (Fig. 3) and low *K*_d_ values for opiate alkaloids in ITC (Fig. 4; Supplementary Table 4). Conversely, the apparent binding affinity of THS was more variable, with some incongruity between ANS displacement and ITC data. For example, whereas ANS displacement yielded no IC_50_ values for thebaine or codeine and a high IC_50_ for morphine (>1 M), ITC data revealed low *K*_d_ values (<20 μM) for all three opiates (Fig. 4; Supplementary Table 4). This disparity could reflect inherent difficulties associated with each technique, particularly when applied to MLP/PR10 proteins. Specifically, if thebaine displaces ANS within the canonical Bet v 1-type hydrophobic binding cavity^53^, the resulting conformational change could expose normally embedded hydrophobic domains that could interact spuriously with ANS and prevent reliable IC_50_ or *K*_d_ calculation. THS-thebaine binding is characterized by a positive entropy factor (ΔS = 28.8 cal/mol/K; Supplementary Table 4), which is sufficiently large to overcome a positive ΔH and allow the interaction to proceed spontaneously. Positive entropy reveals increased disorder and could reflect the emergence of one or more locally unfolded domains after binding or even the requirement of local unfolding for binding to occur^54^. Exposure of ‘intrinsically disordered’ modular domains is typical in the initiation of biomolecular condensates^52^. Even larger positive entropic contributions were noted for PR10-12 upon binding thebaine (39.3 cal/mol/K) and codeine (38.6 cal/mol/K). Notably, the only compound for which negative entropy was observed was the non-alkaloid control ANS (−29.8 cal/mol/K) upon binding MLP15. However, we were unable to resolve the proportion of ΔS resulting from protein conformational change rather than simple ligand exchange entropy or roto-translational entropy^55^. Owing to the poor solubility of some proteins, we experienced inherent difficulties with the broad application of ITC, which precluded reliable calculations for many MLP/PR10-ligand interactions. In cases of low enthalpy (<700 cal/mol), more protein was used to enhance signal-to-noise ratios and achieve estimates of ΔH and *K*_d_ (e.g., PR10-10 and papaverine; Fig. 4).

Structures of the catalytic MLP/PR10 proteins NCS^34^ and THS^33^ have been determined using X-ray crystallography. Herein, crystallographic structures for PR10-10 facilitated a comparison of conformational differences between the *apo* versus *holo* forms. Of particular interest to our corresponding functional data was the detection of a disulfide bond between C155 of chain A and C59 of chain B, linking the *apo* PR10-10 dimers head-to-tail, resulting in the formation of an ‘in-crystal’ polymer. Non-reducing gel electrophoresis of *apo* PR10-10 revealed the presence of disulfide-linked dimers expected under denaturing and non-reducing conditions (Supplementary Fig. 9b). The S-S linkage appears specific to the *apo* form of PR10-10, which, unlike the *holo* enzyme, comprises two structurally distinct protomers distinguished by the presence of a disordered loop in one of the protomers (chain B; Fig. 5 and Supplementary Fig. 8). This disordered loop in the chain B protomer exposes C59, whereas the corresponding residue in chain A protomer is ordered, burying C59. Availability of C59 for disulfide bonding in only one of two *apo* PR10-10 protomers appears to create directionality between the interlocked PR10 protein dimers. Conversely, the PR10-10 dimer bound to papaverine, (*S*)-tetrahydropapaverine, or noscapine is comprised of two structurally identical protomers, each with an ordered loop and buried C59, thus precluding S-S formation between dimer units. The occurrence of other disulfide-mediated oligomers in opium poppy latex has been proposed, including the involvement of enzyme codeinone reductase, the penultimate step in morphine biosynthesis, which has been shown to form disulfide-linked dimers^56^. Since Cys residues are uncommon in the PR10 family^57^, conserved Cys residues in opium poppy MLP/PR10 proteins at positions corresponding to Cys-21, Cys-59 and Cys-155 in PR10-10 support their functional importance in disulfide bond formation. Disulfide bonding has been investigated in other systems as a mechanism affecting protein solubility and aggregate formation. Disulfide bonds have also been shown to modulate aggregate formation in PR10 proteins, including a Der f 2 mutant featuring disrupted S-S bonds, which forms aggregates in the ER and coalesces into unique protein body-like structures^58^. In contrast, mutation of a key cysteine residue in a tree pollen Bet v 1 protein causes distorted and smaller protein body-like aggregates^59^. S-S bonding is potentially a key factor in the ability of opium poppy latex proteins to bind alkaloids and form dense aggregates. Protein aggregate formation could explain how MLP/PR10 proteins are able to support the abundant accumulation of BIAs in latex. Crystallography and ITC results support a 1:1 and 2:1 binding ratio, respectively, which is insufficient to accommodate the direct binding of all accumulating BIA molecules. However, aggregated (unfolded) MLP/PR10 proteins no longer exhibit functioning binding pockets, and instead could form a hydrophobic milieu with the ability to adequately solubilize large quantities of hydrophobic alkaloids.

A direct physiological function for MLP/PR10 proteins in BIA metabolism was demonstrated in a yeast strain engineered to produce opiate alkaloids from the pathway intermediates salutaridine or thebaine. Production of BIAs in engineered microbes is well established^60^, and various yeast strains have been used as tools to discover or validate the function of novel enzymes and transporters^11,23,61–63^. Transient expression of individual MLP/PR10 proteins to engineered yeast resulted in a greater increase in opiate titers when supplied with exogenous salutaridine (Fig. 6). Even a moderate ability to bind hydrophobic and cytotoxic alkaloids, including thebaine (Figs. 3 and 4), appeared to promote greater flux, perhaps by mitigating solubility or toxicity issues involving pathway intermediates or products. The reduced effect of feeding thebaine (Supplementary Fig. 12b, c) could be related to differential cellular uptake. VIGS is an established method to investigate gene function in opium poppy^64^. Previously, strong VIGS-mediated suppression of *NISO* transcript levels decreased the accumulation of morphine, but did not affect the abundance of noscapine or papaverine^24^, although this effect was associated, in part, with the catalytic function of this MLP/PR10 protein. Simultaneous, partial knockdown in the levels of multiple corresponding transcripts affected the actual abundance or the extraction efficiency of morphine, noscapine and papaverine in opium poppy latex (Supplementary Fig. 13b), in support of a physiological role for MLP/PR10 proteins in alkaloid accumulation (Supplementary Information). We cannot rule out the possibility that non-catalytic MLP/PR10 proteins exhibit enzymatic activity on minor alkaloids or transient pathway intermediates, which could (i) reduce product titers in yeast, or (ii) consume alkaloids that are normally channeled into noscapine and papaverine biosynthesis. In the latter scenario, *MLP/PR10* gene silencing could result in the observed increase in papaverine and noscapine levels. Alternatively, the non-catalytic, alkaloid-binding properties of introduced MLP/PR10 proteins could reduce the availability of their associated ligands to BIA pathway enzymes in engineered yeast, resulting in the sequestration of pathway intermediates, such as neopine, and a short-term gain in titer. Similarly, reducing non-catalytic, alkaloid-binding MLP/PR10 protein levels in plants could result in the observed increase in noscapine and papaverine levels.

We used a multifaceted and integrated approach to assess the involvement of MLP/PR10 proteins as auxiliary components involved in BIA metabolism and accumulation. We show that MLP/PR10 proteins interact with BIAs, but the cellular mechanisms underlying their ability to coordinate the accumulation of alkaloids with limited aqueous solubility requires additional investigation, especially with respect to the formation of aggregates or condensates. Our discovery of structural features unique to alkaloid-bound complexes, including S-S bonding, inform future studies regarding the nature and possible contributions of specific MLP/PR10 protein domains and quaternary structures. Our understanding of the metabolic apparatus and product storage in laticifers would benefit from the application of modern microscopic technologies, such as electron cryo-tomography^65^.

## Methods

### Benzylisoquinoline alkaloids

Noscapine hydrochloride and papaverine hydrochloride were obtained from Sigma, USA. Thebaine, oripavine, and morphine sulfate pentahydrate were purchased from Toronto Research Chemicals, Canada. Sources for other alkaloids available from our laboratory collection have been reported previously^66^.

### Plant cultivation

Opium poppy (*Papaver somniferum;* Papaveraceae) plants were cultivated in growth chambers (Conviron, Canada) with long-day photoperiod (16 h at 20 °C/ 8 h at 18 °C) under combination of cool white fluorescent and incandescent lighting^67^. Plant cultivars have been described^32,68^, and Przemko has been further detailed^69^. All research involving controlled materials was performed with appropriate government approvals.

### Sucrose density-gradient fractionation

Sucrose density-gradient fractionation was conducted as shown in Supplementary Fig. 3. Sucrose solutions (66%, 58%, 46%, 38%, 30%, 24 and 14% [w/v]) were prepared in 50 mM HEPES, pH 7.0, containing 5 mM EDTA. Continuous sucrose density gradients were prepared by layering the solutions (3 mL of 66, 58 and 46%, 6 mL of 38%, and 7 mL of 30, 24 and 14% [w/v] sucrose) in 38-mL thin-wall ultracentrifuge tubes (Beckman Coulter, USA) followed by equilibration at 4 °C for 24 h. Fresh latex (80 µL) was collected from decapitated unripe seed capsules in 1.5-mL microcentrifuge tubes and stored on ice for immediate loading on the sucrose gradients. For exogenous alkaloid assays, 15 mg (420 mM) of noscapine hydrochrloide, 12.5 mg (420 mM) of papaverine hydrochloride, or 28 mg (460 mM) of morphine sulfate pentahydrate were added to 80 µL of Przemko latex. Alkaloid quantities were comparable, although differences in the solubilization of various alkaloids in a small volume of latex precluded precise consistency in final concentrations. In all cases, the exogenous alkaloids surpassed naturally observed quantities by 2- to 10-fold, depending on opium poppy variety. For example, exogenous noscapine added to Przemko latex (420 mM) was ~10-fold greater than noscapine content of Roxanne (~40–50 mM)^12,32^. Samples were incubated on ice for 30 min then diluted in 2 mL of latex suspension buffer (500 mM mannitol, 50 mM HEPES, pH 7.0, 5 mM EDTA, 1 mM PMSF) and layered onto sucrose density gradients, which were then centrifuged in an AH-629 rotor (Sorvall, USA) for 3 h at 100,000 × g at 4 °C. The bottom of the ultracentrifuge tube was punctured by a flame-heated 21 G needle and a total of 22 fractions (~1.7 mL) were collected manually. The refractive index of each fraction was measured by a digital refractometer (Reichert, USA) and converted to density. Protein concentration was determined by Bradford assay (BioRad, USA) and samples were stored at −80 °C until use. Fractions were pooled according to density yielding five pooled fractions (ρ = 1.0 to 1.10, 1.11 to 1.14, 1.15 to 1.18, 1.19 to 1.23, and > 1.24 g/mL) and subsequently diluted with sucrose buffer to a final concentration of <15% (w/v) sucrose. Samples were then concentrated in Amicon Ultra-15 10 K centrifugal filters (MilliporeSigma, USA) at 4 °C to a final volume of 500–1000 µL. Sodium dodecyl sulfate (Bioshop, Canada) was added to the concentrated samples to a final concentration of 1% (v/v) and samples were incubated at room temperature for 30 min. The total protein concentration of concentrated samples was estimated using a BCA assay (Thermo Scientific Pierce, USA). Inclusion of SDS to solubilize proteins precluded the use of standard Bradford assays. Proteins were precipitated by adding methanol, chloroform, and water (4:1:3, v/v) and centrifuging at 14,000 × g for 10 min at 4 °C, and protein pellets were washed three times with methanol. The samples were submitted to the Southern Alberta Mass Spectrometry Centre at the University of Calgary (http://sams.ucalgary.ca/) for LC-MS/MS-based shotgun proteomics analysis.

### Sample preparation for proteomics

Protein samples were prepared using a Filter-Aided Sample Preparation (FASP) method. Precipitated proteins (2–200 µg) were resuspended in 230 µL of urea-Tris buffer (8 M urea, 100 mM Tris-HCl, pH 8) containing 10 mM dithiothreitol (Sigma, USA) and incubated at 37 °C for 30 min. Reduced proteins were transferred into Microcon YM-30 (MilliporeSigma, USA) filter units and centrifuged at 14000 × g for 15 min, followed by one wash with 200 µL of urea-Tris buffer. One-hundred microliters of 50 mM iodoacetamide in urea-Tris buffer was added to the filter unit and incubated in the dark at room temperature for 20 min. After centrifugation at 14000 × g for 10 min, samples were washed three times with 100 µL of urea-Tris buffer and again three times with 100 µL of 50 mM ammonium bicarbonate. Protein digestion was performed with a 1:40 ratio of MS-grade trypsin (Promega, USA) to protein in 50 mM ammonium bicarbonate at 37 °C for 16 h. The peptides were collected by centrifugation at 14,000 × g for 10 min, followed by two washes with 40 µL of 50 mM ammonium bicarbonate and one wash with 40 µL of 0.5 M NaCl. Samples were freeze dried and resuspended in 40 µL of 1% (v/v) formic acid for desalting by Peptide Desalting Spin columns (Pierce, USA) according to the manufacturer recommendation.

### LC-MS/MS analysis of peptides

Analysis of tryptic peptides was performed as previously described^23^ with some modifications. Specifically, an Orbitrap Fusion Lumos Tribrid mass spectrometer (Thermo Scientific, USA) operated with Xcalibur (version 4.0.21.10) and coupled to an Easy-nanoflow Liquid Chromatography 1200 system (Thermo Scientific, USA) was used to analyze the peptides. The peptides were loaded onto an Acclaim PepMap 100 C18 trap (75 µm x 2 cm; ThermoScientific, USA) at a flow rate of 2 µL/min of solvent A (0.1% [v/v] formic acid in LC-MS grade water). Peptides were eluted using a 120 min gradient from 5 to 40% (5 to 28% in 105 min followed by an increase to 40% B in 15 min) of solvent B (0.1% [v/v] formic acid in 80% LC-MS grade acetonitrile) at a flow rate of 0.3 µL/min and separated on a PepMap RSLC C18 analytical column (75 um x 50 cm; ThermoScientific, USA). Peptides were electrosprayed at 300 °C using 2.1 kV voltage into the ion transfer tube of the Orbitrap Lumos operating in positive mode. The Orbitrap first performed a full MS scan at a resolution of 120000 FWHM to detect the precursor ion having a *m/z* between 375 and 1575 and a + 2 to +7 charge. The Orbitrap AGC (Auto Gain Control) and the maximum injection time were set at 4e5 and 50 ms, respectively. The Orbitrap was operated using the top speed mode with a 3 sec cycle time for precursor selection. The most intense precursor ions presenting a peptidic isotopic profile and having an intensity threshold of at least 5000 were isolated using the quadrupole and fragmented with HCD (30% collision energy) in the ion-routing multipole. The fragment ions (MS^2^) were analyzed in the ion trap at a rapid scan rate. The AGC and the maximum injection time were set at 1e4 and 35 ms, respectively, for the ion trap. Dynamic exclusion was enabled for 45 sec to avoid of the acquisition of same precursor ion having a similar *m/z* (plus or minus 10 ppm).

### Analysis of proteomics data

The analysis of proteomics data has been previously described^23^. The Lumos raw data files (*.raw) were converted into Mascot Generic Format (MGF) using RawConverter (v1.1.0.18; The Scripps Research Institute) operating in a data dependent mode. Monoisotopic precursors having a charge state of +2 to +7 were selected for conversion. The MGF file was used to search a translated opium poppy latex transcriptome using Mascot algorithm (Matrix Sciences; version 2.4). Mascot uses an ion score whereby an MS/MS match is based on the calculated probability (P) that the observed match between the experimental data and the database sequence is a random event. The reported score is −10Log(P), with conventional selection of 95% confidence. The transcriptome was assembled from RNA-seq data of Roxanne cultivar^68^ (NCBI Sequence Read Archive, BioProject PRJNA508405; Accession number: SRX5557603) as previously described^24^. Specifically, transcriptomes were assembled using the Trinity Assembler35 and associated RSEM (version 1.3.1), R/Bioconductor (version 3.8) packages for transcript abundance estimation. After filtering for redundancy, the global transcriptome included 247,136 transcripts. Search parameters for MS data included trypsin as enzyme, a maximum number of missed cleavages of 1, a peptide charge equal to 2 or higher, cysteine carbamidomethylation as fixed modification, methionine oxidation as variable modification and a mass error tolerance of 10 ppm. A mass error tolerance of 0.6 Da was selected for the fragment ions. Only peptides identified with a confidence score greater than 95% were retained for further analysis. Mascot data files were imported into Scaffold (v5, Proteome Software) for comparison of different samples based on mass spectral counting.

### Preparation of sucrose density-gradient fractions for LC-MS analysis

Sucrose density-gradient fractions (50–100 µL) were diluted 1:1 with acetonitrile and stored at −20 °C. Extracted alkaloids were further diluted with solvent A (10 mM ammonium acetate, pH 5.5; 5% [v/v] acetonitrile) to facilitate linear-range analysis relative to authentic standard curves.

### Production of recombinant MLP/PR10 proteins for binding assays

Opium poppy *MLP/PR10* genes were synthesized (Genscript, USA) with an N-terminal His_6_ tag and subcloned into pACE1. Plasmids were transformed into *E. coli* ArcticExpress (DE3) and protein production was performed using Studier’s Autoinduction system with cultures first grown at 30 °C for 8 h and subsequently left to induce at 16 °C for 24 h. Cells were harvested by centrifugation at 12,000 × g at 4 °C for 20 min. Cell pellets were dried thoroughly, and flash frozen in liquid nitrogen before storage at −80 °C until purification. This process was repeated as needed for the various binding assays. Each cell pellet from a 1-L culture was resuspended in 40 ml EQ solution (50 mM Tris-HCl, pH 7.4, 300 mM NaCl, 10% glycerol [v/v]) containing 50 mg lysozyme, added during a 10-min incubation on ice, followed by sonication on ice for 6 minutes (75% amplitude;10 sec on, 10 sec off). The lysate was clarified by centrifugation at 12 000 × g for 30 min at 4 °C, and then combined with bind 0.5 to 1 ml TALON cobalt-affinity resin (Takara Bio, USA) for 45 min at 4 °C. The resin was allowed to settle for 20 min at 4 °C, and the supernatant was discarded and replaced with fresh EQ buffer. After agitation and resettling of the rinsed resin, the supernatant was again removed, and two additional 20-ml EQ buffer aliquots were added and used to facilitate transfer of the resin into a gravity column (Bio-Rad). Resin wash washed with 10 column volumes of EQ buffer containing 10 mM imidazole and, finally, elution was performed by the stepwise addition of one resin-volume aliquots of EQ buffer containing 200 mM imidazole. Eluates were store separately on ice. For proteins preliminary binding assays, removal of imidazole was achieved by three to four 10-fold dilutions using AmiconUltra 10 kDA ultracentrifugation devices subjected to 3000 × *g* at 4 °C for approximately 20 to 30 min each.

### ANS fluorescence and displacement binding curves

8-anilino-1-naphthalenesulfonic acid (ANS) fluorescence binding curves and the binding-induced reduction in ANS fluorescence resulting from competition with tested ligands across a range of concentration facilitated the measurement of IC_50_ and an estimate of *K*_d_ values using the binding models and procedures described previously^70^. To measure the affinity of each ANS-PR10 interaction and obtain the corresponding *K*_d_ value, binding curves were generated by measuring the fluorescence of a series of assays using a fixed 10 µM concentration of ANS and increasing concentrations of purified recombinant MLP/PR10 proteins (0 to either 80 or 100 µM). ANS was dissolved in dimethylsufoxide at a concentration of 20 mM, stored at −20 °C until dilution and protected from exposure to light. ANS was diluted to 20 µM and individual MLP/PR10 proteins were diluted to various concentrations up to 200 µM in citrate-buffered saline, pH 5.5 (CBS, 10 mM citrate, 127 mM NaCl, 2.7 mM KCl). 40 µl of the ANS solution and 40 µl of each MLP/PR10 protein solution were combined to 80 µl final reaction volumes, which were incubated at 21 °C for 10 min to allow equilibration of binding. Triplicate 18-µl aliquots were withdrawn from each 80 µl reaction, transferred to optically clear PCR tubes (Diamed, Canada) and ANS fluorescence was measured following an additional 3-min incubation at 30 °C using a Qiagen Rotor-gene Q 6-plex with excitation and emission filters of 365 ± 20 nm and 460 ± 20 nm, respectively. An identical reaction series whereby protein was omitted from the mixtures was measured under identical conditions and trace fluorescence was subtracted from each MLP/PR10 protein-containing sample. Each data set was scaled so that the maximum observed fluorescence corresponded to 100% and individual data points were plotted, and a rectangular hyperbolic ligand binding function was fit, using least-squares analysis (Prism 5 software, Graphpad, USA). Experiments were repeated at least twice with independently prepared protein. For PR10-10, the experiment was repeated four times as described above, and twice more with substantially altered MLP/PR10 protein and ANS concentrations.

To measure the ability of major BIAs (i.e., thebaine, morphine, codeine, papaverine and noscapine) to compete with ANS for interaction with the MLP/PR10 proteins, ANS displacement assays were conducted with fixed equimolar concentrations of protein and ANS (10 µM), and a range of BIA ligand concentrations (0 to 3 mM, dependent on solubility). Displacement of ANS is associated with reduced fluorescence. The use of CBS buffer, pH 5.5, as the buffer was required to solubilize papaverine and noscapine. Briefly, 40 µl of 20 µM ANS and individual MLP/ PR10 protein solutions were combined in CBS buffer, pH 5.5, incubated at 21 °C for 5 min, mixed with 40 µl of separate 0 to 6 mM BIA solutions in CBS buffer, pH 5.5, and the 80 µl reactions were subsequently incubated for 10 min at 21 °C. Fluorescence was measured as described above, including subtraction of any fluorescence in a blank series whereby protein was omitted. Individual data points were plotted with fluorescence scaled to 100% and BIA concentrations expressed in their log_10_ form, which allowed fitting of a sigmoidal dose-response model curve and determination of IC_50_ values in Prism 5 software. Each experiment was repeated at least twice with independently prepared MLP/PR10 proteins and BIA ligand. Average IC_50_ values were used, together with corresponding average PR10-ANS *K*_d_ values, to estimate MLP/PR10-BIA *K*_d_ value based on an approximate relationship described previously^71^.

### Isothermal titration calorimetry

ITC experiments were conducted using a Microcal VP instrument (Malvern Pananalytical, Malvern, UK). Recombinant MLP/PR10 protein preparation involving production in *Escherichia coli* followed by cobalt-affinity chromatography was optimized to yield relatively high purity in a single step as determined by SDS-PAGE and colloidal Coomassie blue silver staining. The less than 48 h decrease in atmospheric oxygen exposure generated high-quality proteins with reduced precipitate. To further improve the comparability, proteins were expressed and purified in parallel (e.g., PR10-12 and THS2, NISO and PR10-4, PR10-10 and MLP15) and analyzed without freezing. Eluates were twice dialysed to equilibrium (i.e., 10–12 h at 4 °C) against a 1000-fold (2–4 L) volume of PBS, pH 7.4 (25 mM sodium phosphate, 50 mM NaCl) or CBS, pH 5.5, resulting in a theoretical million-fold dilution of undesired small molecules (e.g., <0.2 µM imidazole). Dialysis was carried out in boiled Spectra/Por 1 6–8 kDa MWCO 0.32 ml/cm (Spectrum Labs, USA) tubing thoroughly washed with either buffer before use. The second 1000-fold volume of dialysis buffer was filtered and degassed using a reusable bottle-top filtration apparatus (Nalgene, USA) with disposable 0.2 µM cellulose filters (VWR, Canada) prior to storage at 4 °C. Ligands were dissolved in the filtered dialysis buffer at 21 °C for approximately one hour, and any insoluble material was removed by repeated centrifugation at 21,000 × *g* for 10 min. No change in pH occurred following substrate addition. Prior to ITC, which was initiated immediately following dialysis, the protein solutions were centrifuged at 17,000 × *g* and 4 °C for 30 min. Any precipitate was separated from the remaining soluble protein fraction which was quantified by absorption at *A*_280nm_ and diluted with filtered dialysis buffer immediately before use.

ITC was performed for THS2, NISO, PR10-4, PR10-10, PR10-12, and MLP15 with ligands suggested by preliminary binding studies or literature reports^23,24^. Thermograms were recorded at 25 °C, with a reference power of 10 (dpu/sec) and monitoring of 30 injections of 9 µl BIA ligand solutions into a sample cell containing 1.4 ml of an MLP/PR10 protein solution, with 300 seconds between injections. Protein concentrations varied from 10 to 150 µM to achieve an optimal C value and curve shape valid for parameter calculation. BIA concentration varied from 0.5 to 3.6 mM based on heat of interaction and solubility. The range of possible ITC experiments were limited by the low solubility of most BIAs in water, notably noscapine and papaverine at pH 7.4, whereas the stability of most MLP/PR10 solutions was relatively low at pH 5.5 owing to the long experimental times, necessary stirring, and extensive degassing. To ensure that we report only the heat of reaction for each interaction, independent titrations of ligand into buffer, and buffer into protein solutions, were used to calculate and then subtract heats of dilution. After subtraction, binding isotherms were analyzed in MicroCal Origin 7 (MicroCal, USA) by fitting a single-site binding model using non-linear least squares regression analysis repeated until convergence. When thermal characteristics of the assay did not conform to expected binding models, fitting was not performed, and parameters were not calculated. Each ITC experiment was repeated 2 to 4 times.

### Production of PR10-10 for X-ray crystallography

Site-directed mutagenesis was used to create a PR10-10 variant, into which a KOD protease site was introduced downstream of the His_6_ tag. Recombinant PR10-10 was produced in the *E. coli* Rosetta 2 strain transformed with a pET47b-PR10-10 expression construct. Starter cultures were grown overnight to an OD_595_ of ~0.4 in 50 ml Luria-Bertani (Miller) broth supplemented with 30 mg/L kanamycin and 35 mg/l chloramphenicol (LBKC) at either 25 or 30 °C with shaking at 170 rpm, and subsequently used to inoculate six 1-L cultures in LBKC broth, which were grown at 37 °C to an OD_595_ of 0.5–0.6 and cooled to 18 °C for 30 min. Isopropyl β-D-1-thiogalactopyranoside was added to a final concentration of 1 mM to induce recombinant protein expression and cultures were incubated at 18 °C for 18–20 h. Cells were then harvested by centrifugation and cell pellets were resuspended in lysis buffer (50 mM sodium phosphate pH 8.0, 10 mM imidazole, 300 mM NaCl, 15% [v/v] glycerol). Resuspended pellets stored at −80 °C were thawed and lysed by sonication in the presence of lysozyme and DNase, and cell debris was subsequently removed by centrifugation at 4 °C. Lysate was loaded onto a 1-ml HisTrap^TM^ HP column (GE Healthcare, USA) and eluted using an imidazole gradient on a BioLogic DuoFlow FPLC (BioRad, USA). Pooled fractions were diluted two-fold in proteolysis buffer (50 mM Bis-Tris-HCl, pH 7.0, 150 mM NaCl, 1 mM EDTA, 1 mM DTT, degassed water) overnight followed by PreScission protease (ThermoFisher, USA) digestion to cleave off the His_6_ tag. GST-tagged protease was removed by running the protein sample through Glutathione Sepharose^TM^ 4B (GE Healthcare, USA) resin. Cleaved protein was dialyzed overnight against the final buffer (20 mM Tris-HCl, pH 8.0, 30 mM NaCl, 2 mM DTT, 0.25 mM EDTA) and spin-concentrated to a final concentration of 15 mg/ml. Concentrated protein was flash-frozen in liquid nitrogen and stored at −80 °C.

### Crystallization and X-ray crystallography

Apo-PR10-10 and PR10-10-alkaloid complexes were crystallized at 15 mg/mL in the presence of 3 mM papaverine and 15% (v/v) methanol, or 2 mM (*R,S*)-tetrahydropapaverine and 10% (v/v) methanol, or with no alkaloid in 12 to 25% (v/v) polyethylene glycol 3350 and 0.1 M Bis-Tris, pH 5.5, via hanging-drop vapor-diffusion at room temperature. Crystallization drops were coated with perfluoropolyether cryogenic oil (Hampton Research, USA), and single crystals were harvested using polymer loops (MiTeGen, USA) and flash-frozen in liquid nitrogen. Crystals were stored in liquid nitrogen until mounted in a nitrogen gas stream at 100 K for diffraction data collection. X-ray diffraction data was measured at the Stanford Synchrotron Radiation Laboratory (SSRL) beamline 12-2 using radiation at a wavelength of 0.98 Å and a Pilatus 6 M pixel array detector (Dectris, Switzerland). Auto XDS^72^ was used for data processing and phases were calculated by molecular replacement using thebaine synthase (56.9% sequence identity, 6KA2) as a search model with PHASER, as implemented in PHENIX^73^. PHENIX was also used for refinement and COOT used for model-building^74^. Molprobity^75^ was used to assess the quality of the models. Coordinates for papaverine and noscapine were obtained from The Cambridge Structural Database (CSD), deposition numbers 107844 and 1306377 respectively. (*S*)-tetrahydropapaverine coordinates were obtained from the crystal structure of Pavine N-methyltransferase complexed with (*S*) and (*R*)-tetrahydropapaverine and adenosylhomocysteine (5KOK)^76^. Restraint files used for refinement were generated using eLBOW as implemented in PHENIX^73^ and proDRG^77^.

### Engineering and growth of yeast strains

Integration cassettes were assembled according to manufacturer’s instructions (Easy clone 2.0 kit^78^, a gift from Irina Borodina; Addgene #1000000073) with modifications. Briefly, coding sequences for BIA biosynthetic genes were codon optimized (GenScript, USA) and subcloned into genome integration vectors using standard, USER-based cloning as descripted previously^24^. Each integrative vector hosted two biosynthesis genes under the control of constitutive, bidirectional *PGK1* and *TDH3* promoters. Five integrative vectors were used to engineer *Saccharomyces cerevisiae* CEN.PK strain: (i) *SalR* at the XI-3 site; (ii) *THS2* and *SalAT* at the XI-5 site; (iii) *T6ODM* and *NISO* at XII-1 site; (iv) *COR-B* and *CODM* at XII-2 site; and (v) *BUP1* at XII-4 site. Alternatively, a NISO variant was inserted with a premature STOP codon 165 bp downstream of the start codon. gRNAs and Cas9-sgRNA expression vectors (https://benchling.com/pub/ellis-crispr-tools#reference) were assembled using the small-fragment golden gate assembly protocol (https://benchling.com/protocols/ACLLwuNs/sgrna-small-fragment-assembly). Prior to transformation, the engineered integrative vectors were subject to linearization by *Not*I digestion (New England Biolabs, USA). Standard LiAc/PEG/ssDNA preparation^79^ was followed for co-transformation of linearized integrative and corresponding Cas9-sgRNA expression vectors. Genomic integration of plasmids into the parent strain was performed in a stepwise manner^80^. Cas9-sgRNA expression vectors carrying selection markers were eliminated by streaking selected colonies on YPD plates.

For transient expression, codon-optimized *PR10* genes containing sequences encoding N-terminus HA tags and flanked by *Sac*II/*Not*I restriction enzyme sites were synthesized (GenScript, USA) and cloned into the pESC-Ura vector. The engineered yeast strain was transformed either with the pESC-Ura vector as the negative control or pESC-Ura vector harboring a gene encoding a PR10 protein driven by the *PGK1* promoter. Individual colonies were used to establish 1.5 ml overnight cultures in SD-Ura medium, and 0.5 ml of each overnight culture was centrifuged, and the medium replaced with fresh SD-Ura medium supplemented with either 50 μM salutaridine or 50 μM thebaine. Next, yeast cells were grown for 2 h or 6 h at 30 °C followed by addition of an equal volume of acetonitrile. Cells were removed by centrifugation, and 5 µl of culture medium was subjected to LC-MS analysis. The remaining 1 ml of overnight yeast culture was centrifuged, and proteins were extracted from the pellet by adding 200 µl of 0.2 M NaOH and incubation for 5 min at room temperature^81^. Total protein extracts (10 µl) from each transformed strain were separated by SDS-PAGE on a 15% (w/v) gel and subjected to immunoblot analysis using anti-HA antibody. HA-tag (GenScript; cat# A01244) primary antibody and goat anti-mouse (BioRad; cat# 170-5047) secondary antibodies were used at dilutions of 1:1000 and 1:20000, respectively.

### Virus-induced gene silencing

VIGS experiments were conducted using opium poppy Bea’s Choice as previously described^64^ with minor modifications. Specifically, a construct was designed with the aim of suppressing a broad range of PR10/MLP family genes (pPR10), targeting highly conserved regions of *MLP15*, *PR10-10*, and *PR10-4* (Supplementary Fig. 14a, Supplementary Data 2). The construct was synthesized and subcloned into the pTRV2-GFP vector using *Xba*I and *Xho*I (GenScript, USA). The pTRV2-GFP vector was modified from pTRV2 by transcriptionally fusing the viral coat protein to GFP at the C-terminal. *Agrobacterium* strain GV3101 carrying either the empty vector pTRV2-GFP (pTRV2), the VIGS construct pPR10 or the pTRV1 helper plasmid were cultured overnight and resuspended in infiltration buffer (10 mM MES, pH 5.6, 10 mM MgCl_2_, 20 μM acetosyringone) to an OD_600_ of 18. Equal volumes of pTRV1 and pTRV2 strains were combined and incubated at room temperature for 2 h. The mixture was infiltrated into the cotyledons and apical meristems of opium poppy seedlings 10 d after germination. Seedlings were grown in a controlled growth cabinet with a 16 h light (20 °C) and 8 h dark (18 °C) photoperiod for a further 30 d before whole seedlings were harvested and stored in liquid nitrogen. Samples were ground to a fine powder, which was then divided in two for alkaloid and gene-expression analysis. Total RNA was extracted using the cetyl trimethyl ammonium bromide method^64^. Genomic DNA was removed from RNA samples using AccuRT Genomic DNA Removal Kit (Applied Biological Materials, Canada) and cDNA synthesis was performed using 1 μg of RNA per sample in a 20 μl reaction using the 5X All-In-One RT MasterMix (Applied Biological Materials, Canada) according to the manufacturers’ instructions. Samples were screened for infection via RT-PCR using the VIGS-CP-F and VIGS-CP-R primers to detect the viral coat protein (Supplementary Fig. 14b; Supplementary Table 6). Infected samples were further analysed by RT-qPCR to determine the relative expression of the *MLP/PR10* transcripts. BIA content was analyzed using LC-MS and compared to corresponding control seedlings infected with the empty pTRV2 vector. qPCR was performed using Power-Up™ SYBR™ Green Master Mix (Applied Biosystems) in a 10-μl reaction containing 200 nM of each primer, and 1 μl of 10-fold diluted cDNA. Three technical replicates were performed for each biological replicate. Thermal conditions on the QuantStudio™ 3 Real-Time PCR System (Applied Biosystems, USA) consisted of 2 hold stages of 50 °C, followed by 95 °C for 2 min each, then by 40 cycles of 95 °C for 1 s and finally 60 °C for 3 s after which a dissociation curve was performed. Gene specific primers were used for all experiments and relative expression was calculated using the 2^-ΔΔCT^ method normalized to the constitutive reference gene glyceraldehyde-3-phosphate dehydrogenase (Supplementary Table 6).

Prior to processing for alkaloid analysis, frozen ground tissue was lyophilized, then weighed. Alkaloids were extracted with 400 μl of a 1:1 (v/v) solution of acetonitrile:methanol for 4 h at room temperature on an orbital shaker. Samples were centrifuged at 13,000 x g for 20 min then 4 μl of supernatant was transferred to a fresh microfuge tube containing 196 ul of acetonitrile: methanol (1:1 [v/v]). Samples were centrifuged for an additional 10 min at 13,000 × *g* and 150 μl of the supernatant was added to glass vials, and 10 μl was used for LC-MS for analysis in full-scan mode. To determine the statistical significance of VIGS gene expression and metabolite data, Student’s *t*-test was performed (unpaired, two-tailed) using Prism 5 software.

### LC-MS analysis

Alkaloid analysis was conducted using a 6410 triple quadrupole LC-MS (Agilent Technologies, USA). Samples (10 µL) were injected into the LC-MS/MS system and separated using a Poroshell 120 SB-C18 HPLC column (Agilent Technologies, USA) with a flow rate of 0.6 ml/min and a gradient of solvent A and solvent B (100% acetonitrile): 0–60% (v/v) solvent B from 0 to 8 min, 60–99% (v/v) solvent B from 8 to 10 min, isocratic 99% (v/v) solvent B from 10 to 11 min, 99–0% (v/v) solvent B from 11 to 11.1 min, 0% solvent B from 11.1 to 14.1 min. The analyses were performed in positive electrospray ionization (ESI) mode (gas temperature, 350 °C; gas flow rate, 10 L/min; nebulizer gas pressure, 50 psi; capillary voltage 4000 V), scanning with a mass range of 200–700 *m/z*. Data were processed and analyzed using Agilent MassHunter Qualitative Analysis software (version B.06.00). Using full-scan data, extracted ion chromatographs were used for peak area determination. Comparison of analytes with the retention times (R_t_) and collision-induced dissociation (CID) spectra of authentic standards was used to identify alkaloids. Quantification was performed using standard curves of authentic standards.

### Reporting summary

Further information on research design is available in the Nature Research Reporting Summary linked to this article.

## Supplementary information

Supplementary Information

Description of Additional Supplementary Files

Supplementary Data 1

Supplementary Data 2

Reporting Summary

## Data Availability

The proteomics datasets are deposited in the ProteomeXchange Consortium (http://proteomecentral.proteomexchange.org) via the PRIDE partner repository with the dataset identifier PXD035860. The structures determined by X-ray crystallography are available in the Protein Data Bank (https://www.rcsb.org) under the accession codes 7UQL, 7UQM, 7UQN, and 7UQO. A Source Data file is provided. Source data are provided with this paper.

## Source data

Source Data
