# Supplementary material for: Alkaloid binding to opium poppy major latex proteins triggers structural modification and functional aggregation
Source: Nat Commun. 2022 Nov 9;13:6768. doi: 10.1038/s41467-022-34313-6 (PMC9646721; doi:10.1038/s41467-022-34313-6)
Supplement: Supplementary file 1 — Supplementary Information [file 41467_2022_34313_MOESM1_ESM.pdf]

Supplementary Discussion

Sucrose density gradient fractionation of opium poppy latex

To further explore the nature of dense aggregate within poppy extracts, alkaloid-free latex harvested from Przemko poppies was mixed with exogenous alkaloids prior to density gradient fractionation (Fig. 2). Although Przemko latex contains the full complement of known BIA biosynthetic enzymes and PR10 proteins, it is devoid of alkaloids, and routine fractionation did not reveal any high-density aggregates (Fig. 1). Addition of morphine to Przemko latex did not visibly form this flocculent material; however, upon closer inspection, morphine was dramatically elevated in higher-density fractions compared to controls where no latex was present (Supplementary Fig. 6). This result coincided with a subtle but notable shift of latex protein downward in the gradient (compare Fig. 1 with Fig. 2). More dramatic results were obtained upon fractionating papaverine or noscapine with latex, where one or more visibly insoluble dense aggregate bands appeared (Fig. 2). Control runs revealed that both papaverine and noscapine distribute along the gradient when no latex protein is present (Supplementary Fig. 6). However, the distribution pattern is altered when both components (latex + alkaloid) are included, and both papaverine and noscapine become more abundant in higher-density fractions aligned with (1) the appearance of insoluble bands and (2) shifts in in enzyme and/or PR10 protein localization along the gradient (Fig. 2).

ANS displacement and isothermal titration calorimetry

Certain features of 8-anilino-1-naphthalenesulfonic acid (ANS) displacement and isothermal titration calorimetry (ITC) methodologies as applied to latex PR10s rendered analysis difficult and/or posed caveats precluding accurate parameter determination. For example, should thebaine successfully displace ANS within the canonical Bet v 1-type hydrophobic binding cavity[1], it is not inconceivable that the resulting conformational change would expose normally embedded hydrophobic domains that could interact spuriously with ANS and prevent reliable Ki calculation. THS-thebaine binding is characterized by a positive entropy factor (ΔS = 28.8 cal/mol/K; Supplementary Table 4) which is sufficiently large to overcome a positive (endothermic) ΔH and allow the reaction to proceed spontaneously. ITC, as applied herein, was met with difficulties keeping protein in solution, precluding reliable calculations for many PR10-ligand interactions (Supplementary Table 4). In cases of low enthalpy (<700 cal/mol), increased protein was deployed to enhance signal-to-noise ratios and achieve estimates of ΔH and K_D (e.g., PR10-10 and papaverine; Fig. 5). However, in many cases, this approach exacerbated precipitation problems. Protein solubility and aggregation are common issues associated with ITC[2].

Under standard ITC conditions using up to 120 µM PR10-10 or 110 µM MLP15 and various ligands at concentrations up to their solubility threshold (i.e., 1.2 mM for papaverine and 1.1 mM for noscapine at pH 5.5), reliable binding curves were difficult to establish. However, the optimization of control experiments, including injections of ligand into buffer-only filled sample cells and injections of the buffer without ligand into protein-solution filled sample cells (each revealing a minor heat of dilution) showed that both PR10-10 and MLP15 caused a substantial change in the thermodynamic properties of the system in a manner consistent with binding. Whereas injections of papaverine into a solution containing only buffer produced a thermogram with endothermic peaks (0.25 µcal/sec), injection of papaverine into buffer containing PR10-10 produced thermograms with (exothermic) peaks of the opposite sign, which suggested a protein-ligand interaction. Subtraction of the blank value showed that the PR10-10-papaverine interaction caused heat changes reaching -0.2 µcal/sec. Although the modest change in peak

areas precluded an ideal curve fit, a K_d of 225 µM was estimated. Similarly, injection of noscapine into a sample cell containing only a buffered solution produced a thermogram with endothermic peaks that reached nearly 0.9 µcal/sec. Injections of noscapine into a sample cell containing buffer and MLP15 produced an unusual thermogram whereby each injection yielded two peaks or heat-change events slightly separated over time. During the initial injections, the first and endothermic heat-change predominated and positive peaks reaching >0.2 µcal/sec were detected. Intermediate injections showed two slightly overlapping peaks, which essentially canceled each other during integration. In contrast, the final injections eventually produced a thermogram dominated by the second, exothermic heat-change event that plateaued at -0.48 µcal/sec. Curves corresponding to single-site, multiple-site, and sequential binding-site models could not be reliably fit to the data after subtracting the blank, which represented the heat of dilution of noscapine. Since the nature of the two heat-change events could not be determined, and a theoretical basis for the data could not be established, binding affinity was not calculated.

PR10-10 crystal structure

Apo PR10-10 was refined to a resolution of 1.90Å from $P2_12_12$ crystals with two copies (A and B) in the asymmetric unit that form a homodimer. The following residues did not show sufficient electron density to be modeled: (i) N-terminal residues 1-7 from chain A and 1-6 from chain B, (ii) residues 37-40 from chains A, 47-57 for chain B, and (iii) C-terminal residues 156-158 from chain A and 157-158 from chain B. PR10-10 papaverine, (*S*)-tetrahydropapaverine, and noscapine complexes were refined to a resolution of 1.75Å, 1.79Å, and 1.70Å, respectively, from $C222_1$ crystals with one copy in the asymmetric unit. The following residues from the PR10-10-papaverine complex did not show sufficient electron density to be modeled: (i) C-terminal residues 1-7, (ii) residues 34-37, (iii) residues 52-53, (iv) residues 114-115, and (v) C-terminal residues 157-158. The following residues from the PR10-10-(*S*)-tetrahydropapaverine complex did not show sufficient electron density to be modeled: (i) N-terminal residues 1-7, (ii) residues 34-41, and (iii) C-terminal residues 157-158. The following residues from the PR10-10-noscapine complex did not show sufficient electron density to be modeled: (i) N-terminal residues 1-9, (ii) residues 35-36, and (iii) C-terminal residue 158.

Immediately apparent was a disulfide bond formed between C155 of chain A and C59 of chain B, essentially linking the *apo* PR10-10 dimers head to tail to form an “in-crystal” polymer. The *S*-*S* linkage appears entirely specific to the *apo* form of PR10-10, which unlike the *holo* enzyme is comprised of two structurally distinct dimer units (protomers) distinguished by the presence of a disordered loop in one of protomers. This disordered loop in the chain B protomer features an ‘exposed’ C59, whereas the corresponding amino acid sequence in chain A protomer is ordered, forcing C59 to be buried. Conversely, PR10-10 dimer bound to papaverine, tetrahydropapaverine or noscapine is comprised of two structurally identical protomers, each with an ordered loop and buried C59, thus precluding *S*-*S* formation between dimer units. The *S*-*S* linkages are directional, as chain A contributes C155 and chain B contributes C59, exclusively providing directionality. Specifically, C59 from chain A is buried by the a1b3 loop (the region with disordered b2 strand), making C59 inaccessible for disulfide formation, whereas C155 from chain B is in the same conformation as in chain A (disulfide forming C155) but does not form a disulfide.

The noscapine-PR10-10 complex is somewhat more ambiguous compared to the papaverine- and (*S*)-tetrahydropapaverine-PR10-10 complexes. Electron density in the alkaloid-binding pocket is less clear and did not show much improvement during refinement. Despite the relatively weaker electron density, the density can be confidently interpreted to represent a bound noscapine molecule. Trp63 adopts the *holo* PR10-10 conformation, since it is rotated out of the alkaloid binding pocket, consistent with papaverine- and (*S*)-tetrahydropapaverine-PR10-10 complexes (Fig. 5c-d). Additionally, crystals of the noscapine-, papaverine- and

tetrahydropapaverine-PR10-10 complexes all show the same crystal from, $C222_1$; which differs from *apo* PR10-10 crystals, $P2_12_12$. The relatively weaker electron density for noscapine suggests a more flexible binding state, analogous to the isoquinoline moieties of papaverine and (*S*)-tetrahydropapaverine.

Sequence alignments and structural comparison

The structures of two PR10-like enzymes acting on benzylisoquinoline alkaloid (BIA) substrates have previously been solved: *Papaver somniferum* THS (6KA2, 51.1% sequence identity, 1.2 Å root mean square deviation (RMSD)[3] and *Thalictrum flavum* NCS (2VQ5, 13.4% sequence identity, 5.6 Å RMSD)[4]. The comparison of PR10-10 and NSC (2VQ5) reveals conservation of the Bet v 1-fold but showed little structural and sequence conservation in the binding pocket. In contrast, the superposition of PR10-10 and THS (6KA2) demonstrates strong conservation of residues in the alkaloid binding pocket (Supplementary Fig. 11). Additionally, THS and PR10-10 show the same homodimeric structure (1.4 Å RMSD), which differs from the NCS tetramer dimer units. A key difference is F143 in PR10-10, which corresponds to G143 in THS. F143 from PR10-10 clashes with thebaine in superpositions of PR10-10 and THS, whereas G143 widens the binding pocket. Sequence alignments of PR10-10 with related members of the opium poppy latex PR10/MLP family show that position 143 in PR10-10 is conserved as a phenylalanine in most PR10s except for the substitution of a cysteine in PR10-11 and MLP2, valine for PR10-12 and PR10-5, tyrosine for MLP4, and glycine in THS (Supplementary Fig. 11). Positions 63, 76, and 89 are conserved across all MLP/PR10 proteins as tryptophan, glutamine, and histidine, respectively. W63 in both PR10-10 and THS likely mediate hydrophobic interactions, staggered in PR10-10 and perpendicular in THS[3], with bound alkaloids. Although W63 adopts similar conformations in PR10-10 and THS, the residue is more retracted from the binding pocket in PR10-10, which makes the pocket larger on that respective side (Supplementary Fig. 11). Widening of the PR10-10 binding pocket results from the β-sheet adopting a wider conformation in the complexed form versus the *apo* form. The strict conservation, hydrophobic nature, and central positioning of W63 in the alkaloid binding pocket suggest that this residue is crucial for alkaloid binding. E76 likely forms hydrogen bonds with the nitrogen atom of papaverine, noscapine, and (*S*)-tetrahydropapaverine mediated by a water molecule. Although E76 is conserved in THS, the proposed interaction of the residue with the bound alkaloid is not conserved. The nitrogen from thebaine in THS is located within hydrogen bonding distance of S74[3], which is not conserved in PR10-10 or other PR10/MLP proteins. Nevertheless, the conservation of E76 and its apparent function in PR10-10 suggests an important role for this residue in interactions with the polar and/or charged regions of bound alkaloids. Notably, in all four available complexed BIA-PR10 structures, the nitrogen group of the alkaloid exhibits a charged or polar residue within hydrogen bonding distance, specifically S74 in THS and E76 in PR10-10. This observation may assist with the modeling of other PR10 complexed structures. H89 and T105 have been proposed as catalytic residues in THS[3]. H89 is strictly conserved across the PR10 and MLP family, while T105 shows considerable variability, being substituted by valine in PR10-10.

VIGS

Strong phenotypic modulations have been achieved through VIGS-mediated knockdown of transcript encoding single BIA biosynthetic enzymes[5–8]. Although multiple genes can be co-suppressed by targeting conserved regions, the simultaneous reduction of related transcript levels generally results in weaker phenotypic changes[9]. Thus, a partial knockdown of only MLP/PR10 transcripts and the limited alteration in BIA content was expected (Supplementary Fig. 13). Sequence similarity among transcripts limited the effectiveness of VIGS in mediating a

systematic reduction in the levels of individual, functionally redundant MLP/PR10 proteins in the latex.

Supplementary references

1. Mogensen, J. E., Wimmer, R., Larsen, J. N., Spangfort, M. D. & Otzen, D. E. The major birch allergen, Bet v 1, shows affinity for a broad spectrum of physiological ligands. *J. Biol. Chem.* **277**, 23684–23692 (2002).
2. Tellinghuisen, J. Designing isothermal titration calorimetry experiments for the study of 1:1 binding: Problems with the 'standard protocol'. *Anal. Biochem.* **424**, 211–220 (2012).
3. Chen, C. C. *et al.* Structural insights into thebaine synthase 2 catalysis. *Biochem. Biophys. Res. Commun.* **529**, 156–161 (2020).
4. Ilari, A. *et al.* Structural basis of enzymatic (*S*)-norcoclaurine biosynthesis. *J. Biol. Chem.* **284**, 897–904 (2009).
5. Hagel, J. M. & Facchini, P. J. Dioxygenases catalyze the *O*-demethylation steps of morphine biosynthesis in opium poppy. *Nat. Chem. Biol.* **6**, 273–275 (2010).
6. Farrow, S. C., Hagel, J. M., Beaudoin, G. A. W., Burns, D. C. & Facchini, P. J. Stereochemical inversion of (S)-reticuline by a cytochrome P450 fusion in opium poppy. *Nat. Chem. Biol.* **11**, 728–732 (2015).
7. Chen, X. *et al.* A pathogenesis-related 10 protein catalyzes the final step in thebaine biosynthesis article. *Nat. Chem. Biol.* **14**, 738–743 (2018).
8. Dastmalchi, M. *et al.* Neopinone isomerase is involved in codeine and morphine biosynthesis in opium poppy. *Nat. Chem. Biol.* **15**, 384–390 (2019).
9. Dastmalchi, M. *et al.* Purine permease-type benzylisoquinoline alkaloid transporters in opium poppy. *Plant Physiol.* **181**, 916–933 (2019).

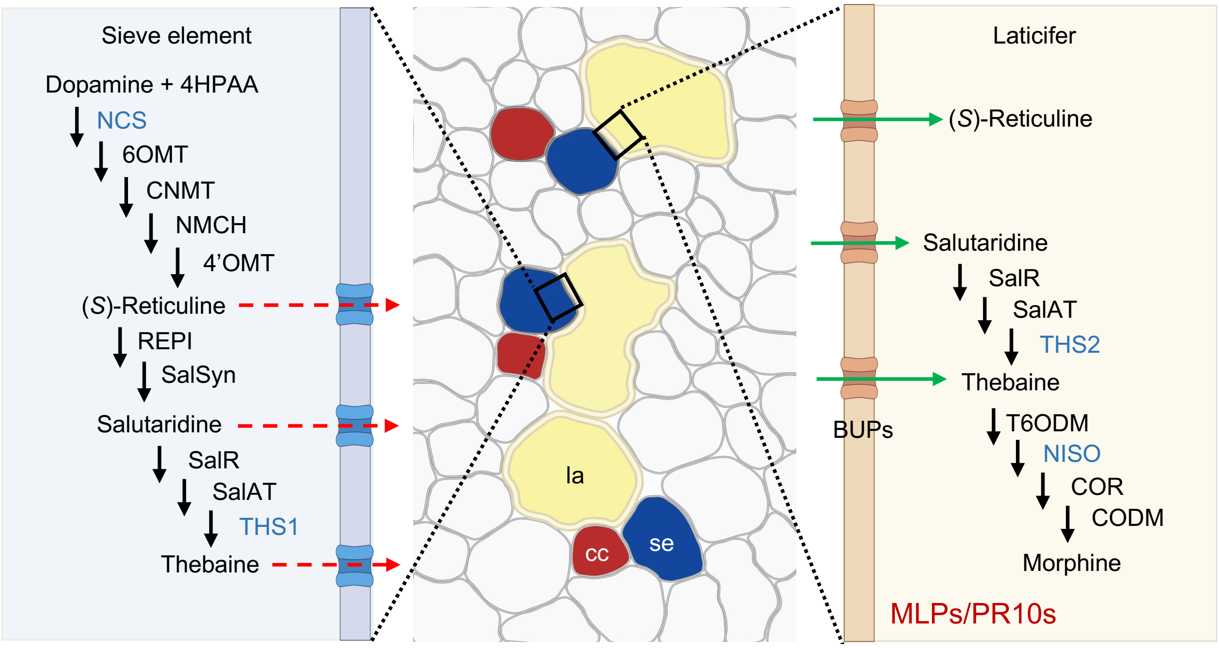

Supplementary Figure 1. Model showing the localization of morphine biosynthesis in opium poppy. Three cell types are involved in the biosynthesis and accumulation of morphine in opium poppy: companion cells (cc, red), sieve elements (se, blue), and laticifers (la, yellow). Genes encoding biosynthetic enzymes are expressed in companion cells with the cognate proteins translocated to sieve elements, or in laticifers with the corresponding proteins remaining in the latex. The initial steps of morphine biosynthesis up to salutaridine are restricted to sieve elements, whereas conversion of salutaridine to thebaine occurs in both sieve elements and laticifers. The final steps in the transformation of thebaine to morphine are predominant in laticifers. Morphine pathway intermediates are translocated from sieve elements to laticifers via export (red dashed arrows) and import proteins, including a family of benzylisoquinoline uptake permease transporters (BUPs) with a broad substrate range and associated with laticifers (green arrows). Dashed arrows represent unknown export mechanism. Enzymes belonging to the MLP/PR10 protein family are shown in blue. Latex MLP/PR10 proteins are depicted in red. 4′OMT, 3′-hydroxy-*N*-methylcoclaurine 4′-*O*-methyltransferase; 6OMT, norcoclaurine 6-*O*-methyltransferase; CNMT, coclaurine *N*-methyltransferase; CODM, codeine *O*-demethylase; COR, codeinone reductase; NCS, norcoclaurine synthase; NISO, neopinone isomerase; NMCH, *N*-methylcoclaurine 3′-hydroxylase; REPI, reticuline epimerase; SalAT, salutaridinol 7-*O*-acetyltransferase; SalR, salutaridine reductase; SalSyn, salutaridine synthase; T6ODM, thebaine 6-*O*-demethylase; THS, thebaine synthase. Created with BioRender.com.

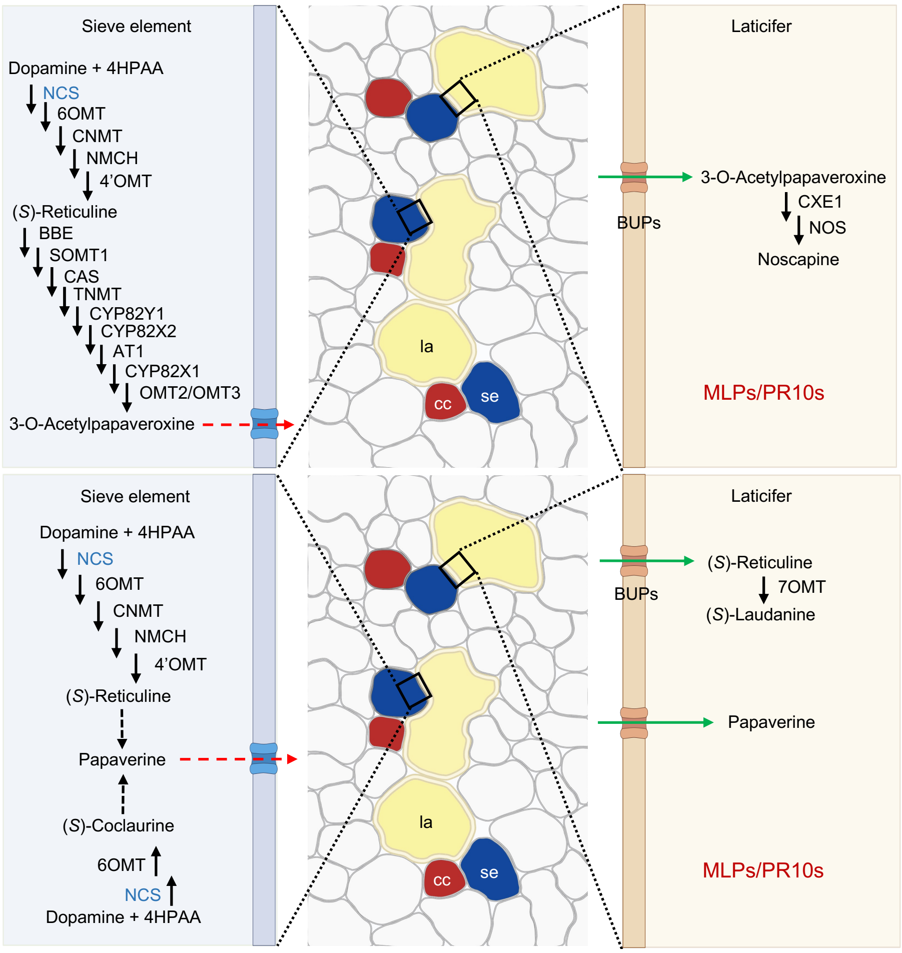

Supplementary Figure 2. Model showing the localization of noscapine and papaverine biosynthesis in opium poppy. Three cell types are involved in the biosynthesis and accumulation of noscapine (upper panel) and papaverine (lower panel): companion cells (cc, red), sieve elements (se, blue), and laticifers (la, yellow). Papaverine biosynthetic genes, and most noscapine biosynthetic genes (up to the intermediate 3-*O*-acetylpapaveroxine), are expressed in companion cells with the cognate proteins translocated to sieve elements. The last two genes in the noscapine pathway are expressed in laticifers with the corresponding proteins remaining in the latex. Pathway intermediates and products are translocated from sieve elements to laticifers via export (red dashed arrow) and import proteins, including a family of benzylisoquinoline uptake permease transporters (BUPs; green arrows). Dashed arrows represent unknown biosynthetic steps or transport mechanisms. Enzymes belonging to the MLP/PR10 protein family are shown in blue. Latex MLP/PR10 proteins are depicted in red. Abbreviations: 4′OMT, 3′-hydroxy-*N*-methylcoclaurine 4′-*O*-methyltransferase; 4-HPAA, 4-hydroxyphenylacetaldehyde; 6OMT, norcoclaurine 6-*O*-methyltransferase; 7OMT, reticuline 7-*O*-methyltransferase; AT1, 1,13-dihydroxy-*N*-methylcanadine 13-*O*-acetyltransferase; BBE, berberine bridge enzyme; CAS, canadine synthase; CNMT, coclaurine *N*-methyltransferase; CXE1, 3-*O*-acetylpapaveroxine carboxylesterase; CYP82X1, 1-hydroxy-13-*O*-acetyl-*N*-methylcanadine 8-hydroxylase; CYP82X2, 1-hydroxy-*N*-methylcanadine 13-*O*-hydroxylase; CYP82Y1, *N*-methylcanadine 1-hydroxylase; NCS, norcoclaurine synthase; NMCH, *N*-methylcoclaurine 3′-hydroxylase; NOS, noscapine synthase; OMT2:OMT3, 4′-*O*-desmethyl-3-*O*-acetylpapaveroxine 4′-*O*-methyltransferase; SOMT, scoulerine 9-*O*-methyltransferase; TNMT, tetrahydroprotoberberine *N*-methyltransferase. Created with BioRender.com.

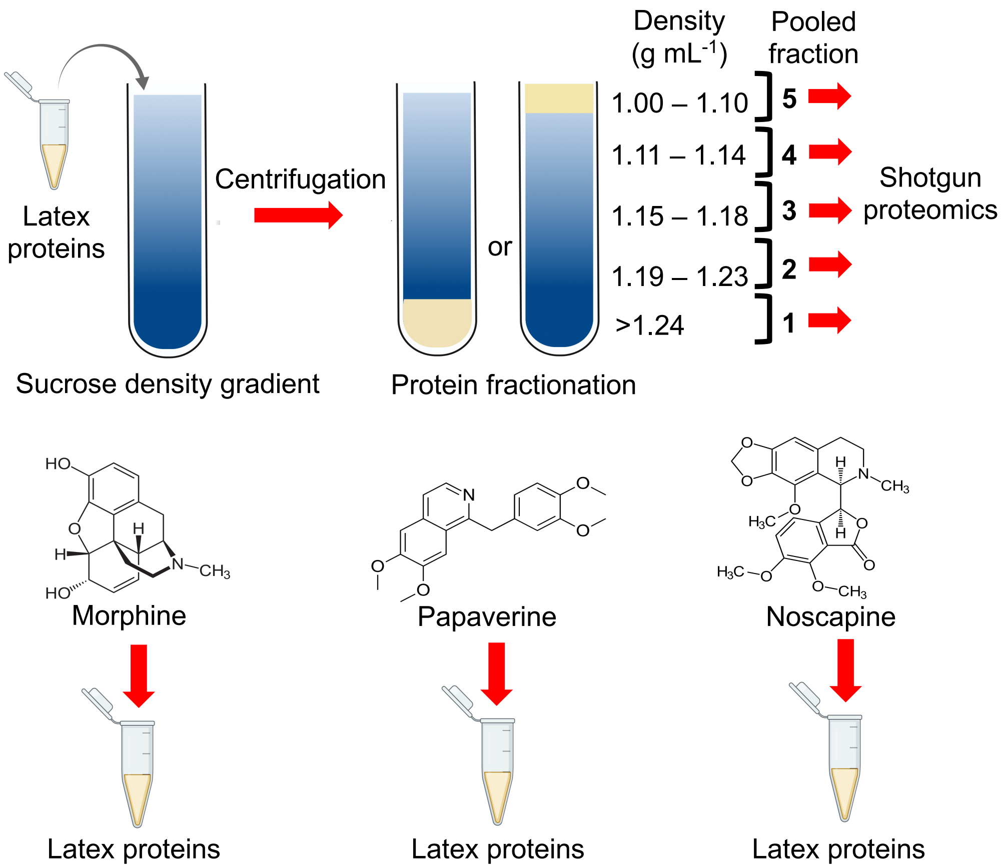

Supplementary Figure 3. Schematic representation of the protocols used for sucrose density-gradient fractionation of opium poppy latex. Opium poppy latex was fractionated on a 14-66% (w/v) sucrose density gradient (upper panel). After ultracentrifugation, 22 fractions (each ~1.7 ml) were collected and subjected to alkaloid profiling by LC-MS/MS. Fractions were pooled according to density (ρ = 1.0 – 1.10, 1.11 – 1.14, 1.15 –1.18, 1.19 – 1.23, and >1.24 g/mL) and pooled fractions were analyzed by shotgun proteomics. Exogenous morphine, papaverine or noscapine was added to latex from the Przemko chemotype (lower panel) and sucrose-gradient analysis was carried out as in the upper panel. Created with BioRender.com.

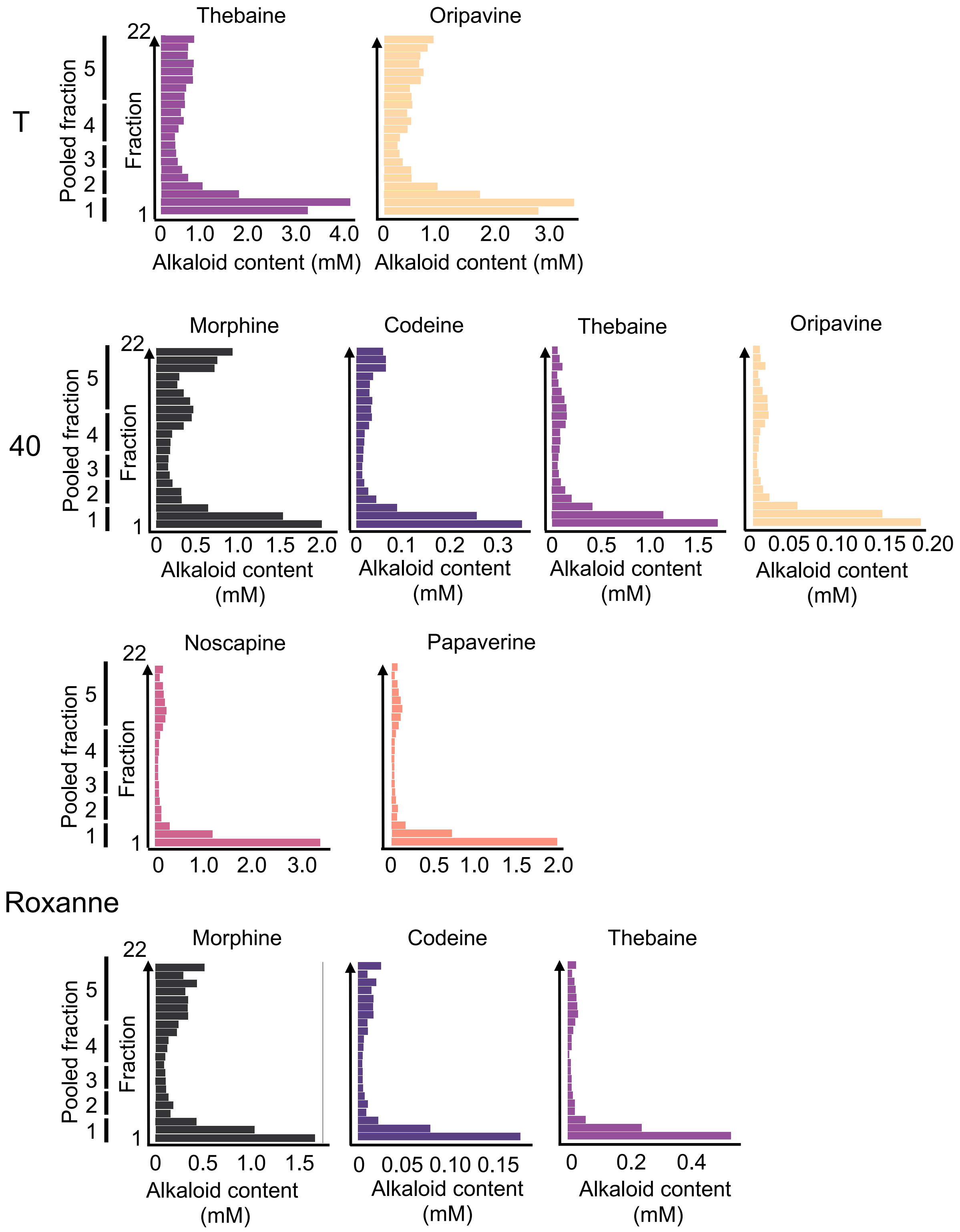

Supplementary Figure 4. Alkaloid analysis of sucrose density-gradient fractions. Abundance of major alkaloids in sucrose density-gradient fractions of opium poppy latex from the chemotypes T, 40, and Roxanne. Note that pooled fractions 1-5 (Fig. 1) are comprised of unpooled fractions 1-22.

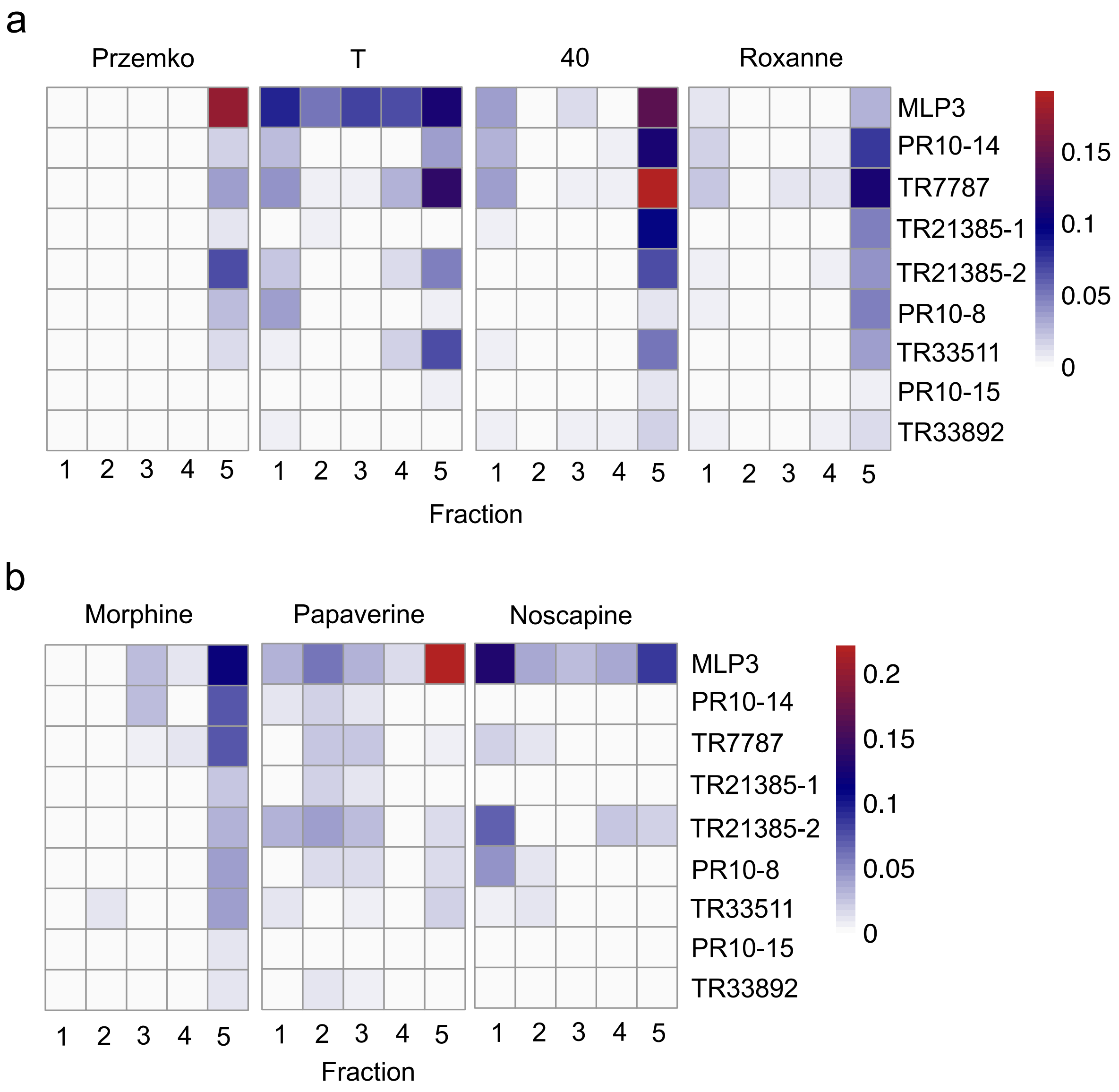

Supplementary Figure 5. Heat maps showing the relative abundance of minor MLP/PR10 proteins in pooled fractions. a, Latex from opium poppy chemotypes Przemko, T, 40 and Roxanne. **b,** Przemko latex containing exogenous morphine, papaverine and noscapine. The color scale indicates percentage of total spectra. Color scales indicating percentage of the total spectral counts in each proteome are shown on the right side of the panels. Note that pooled fractions 1-5 (shown here) are comprised of unpooled fractions 1-22 (e.g. Supplementary Fig. 4, 6)

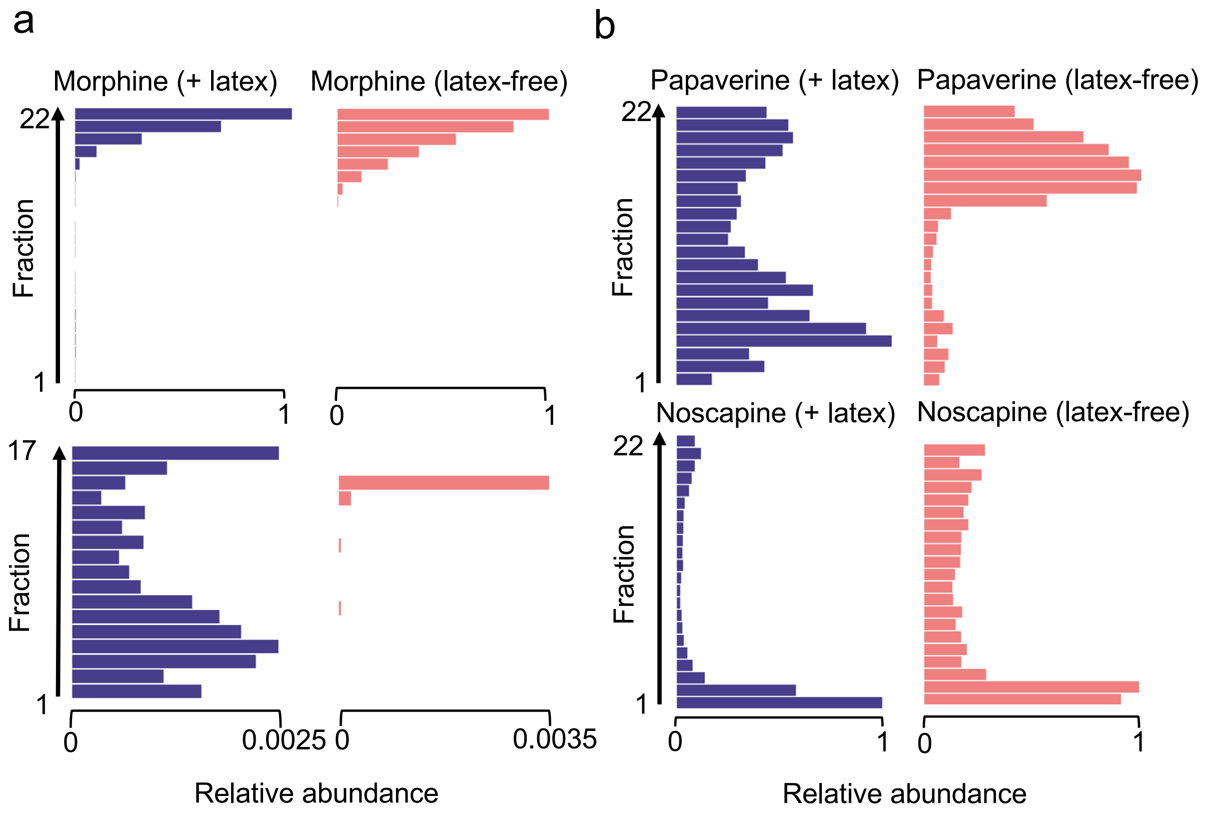

Supplementary Figure 6. Alkaloid analysis of latex-free, sucrose density-gradient fractionated pure morphine, papaverine and noscapine. a, morphine, **b**, papaverine and noscapine abundance in gradient fractions in the absence (coral) or presence of latex (dark purple; Fig. 2a). The bottom graph of **a** represents magnified fractions (1-17, latex; 1-15, latex-free) from the upper graph. Note that pooled fractions 1-5 (Fig. 1) are comprised of unpooled fractions 1-22.

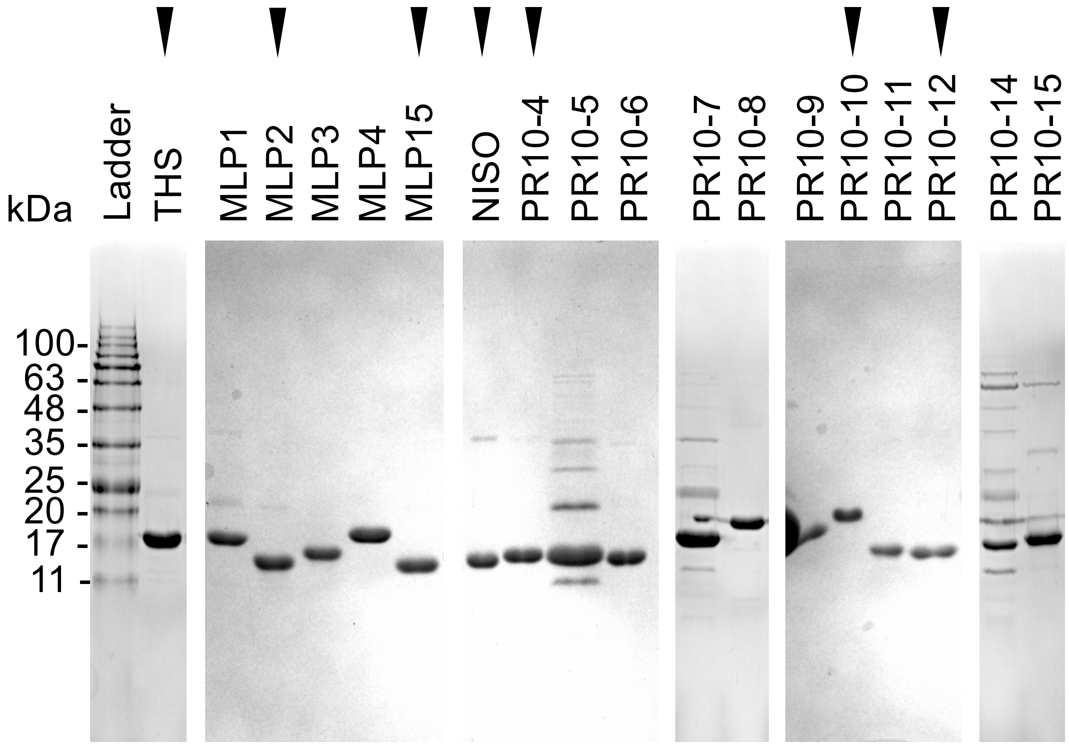

Supplementary Figure 7. SDS-PAGE analysis of recombinant, cobalt-affinity purified MLP/PR10 proteins. Each sample contained 2 µg of protein and the gel was stained with Coomassie R-250. Arrowheads indicate proteins most abundant in opium poppy latex and selected for ligand-binding analyses. The experiment was repeated twice with similar results.

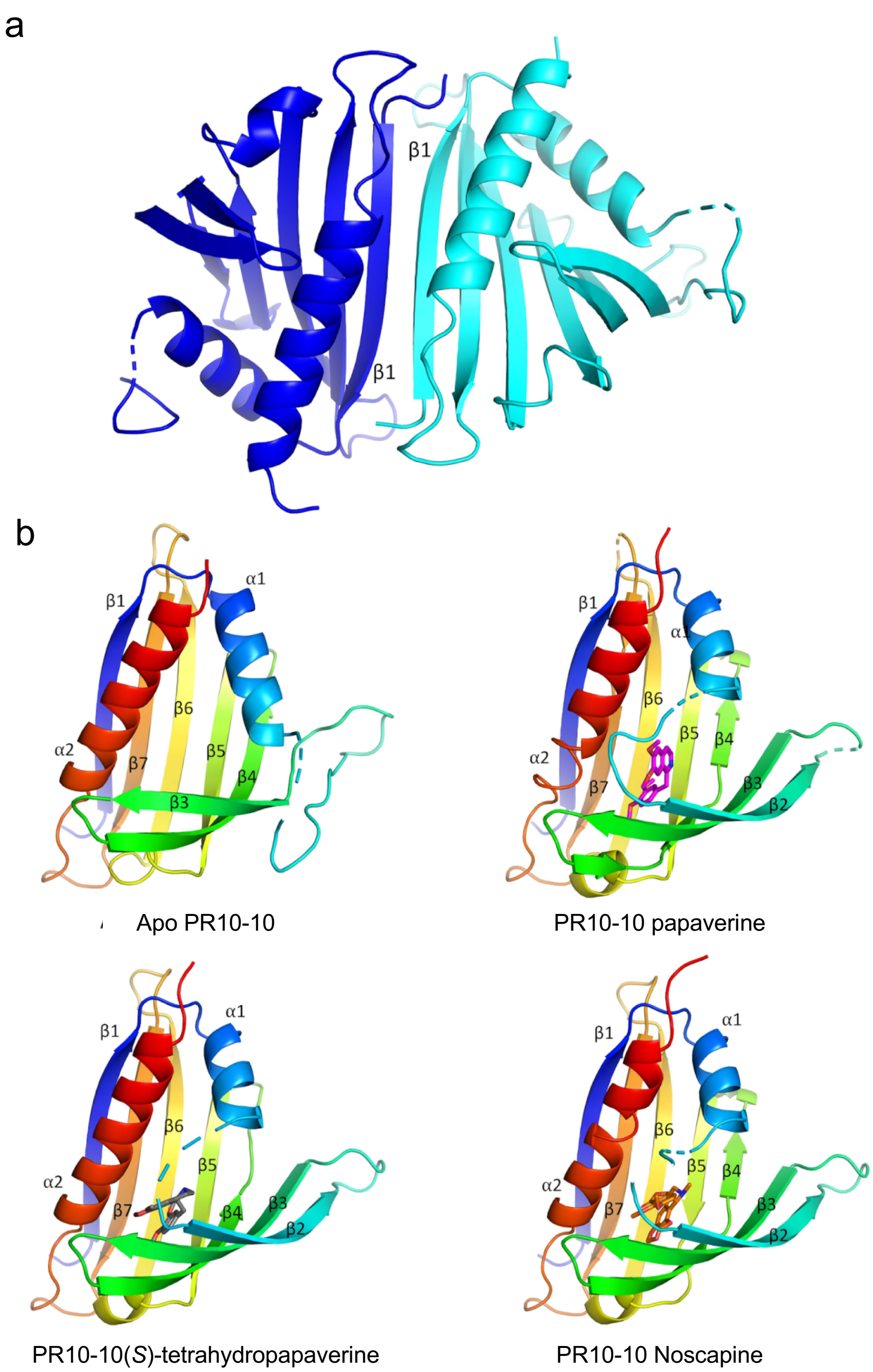

Supplementary Figure 8. Additional views of the crystal structure of PR10-10 dimer and complexes with noscapine, papaverine, and (*S*)-tetrahydropapaverine. a, Front view of the Apo PR10-10 dimer (note that complexed PR10-10 forms the same dimer). Protomers are shown in cyan and blue and only the α-carbon backbone drawn. **b**, Front view of single PR10-10 protomers for the *apo* and bound (papaverine, (*S*)-tetrahydropapaverine, and noscapine) structures of PR10-10. The rainbow coloring scheme starts at the C-terminus in red to the N-terminus in blue. Helices and β strands are numbered accordingly. Papaverine is shown in magenta, (*S*)-tetrahydropapaverine is shown in grey, and noscapine is shown in orange.

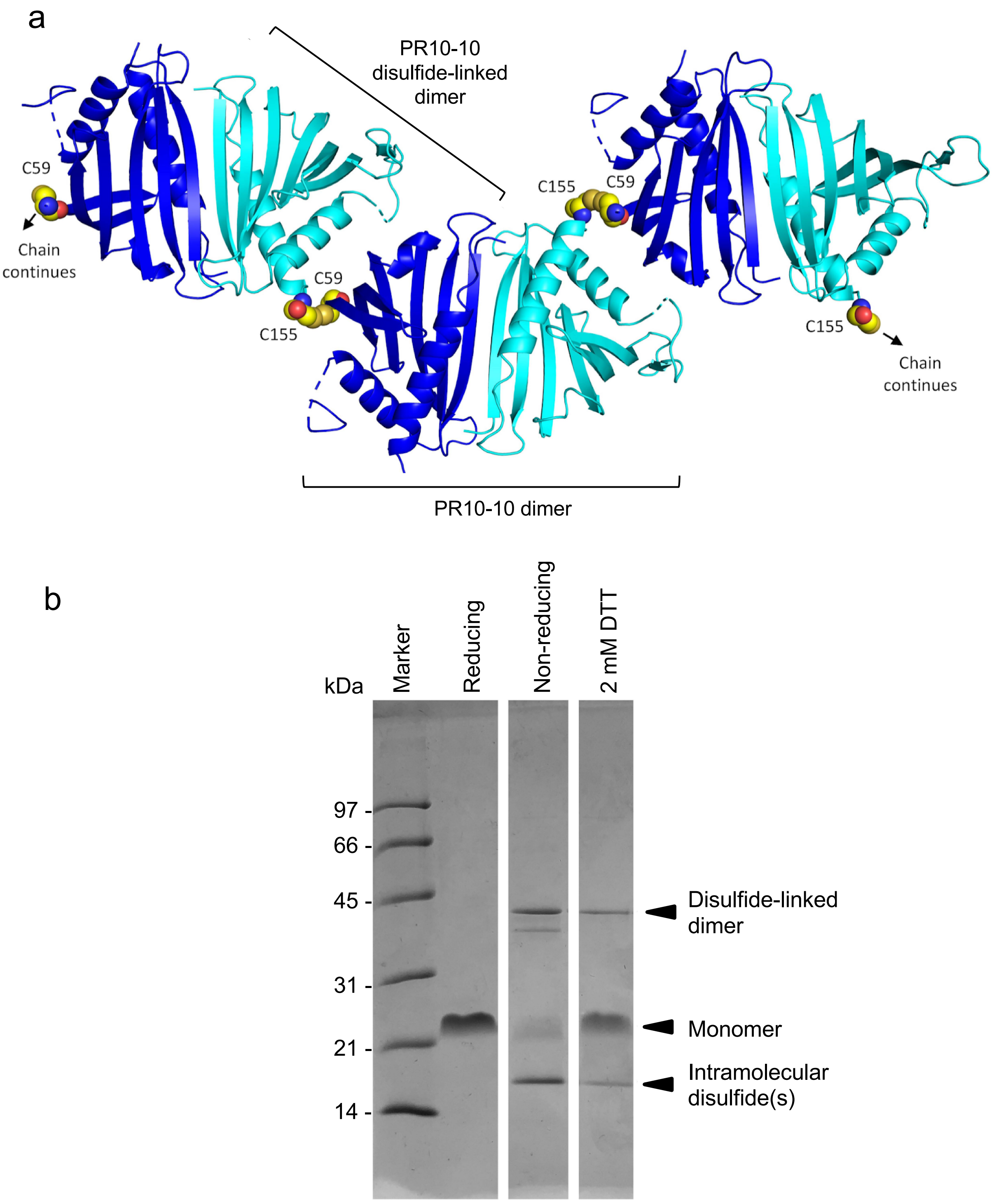

Supplementary Figure 9. Oligomeric state of Apo PR10-10. Disulfide mediated PR10-10 polymers. **a**, Apo PR10-10 “in-crystal” polymer. Protomers from the PR10-10 dimer are shown in cyan and blue. Disulfides are shown in yellow spheres. **b**, Reducing and non-reducing SDS-PAGE analysis of Apo PR10-10. β-mercaptoethanol was used for reducing conditions and 2mM dithiothreitol (DTT) was tested to better represent the crystallization conditions. Gels were stained with Coomassie R-250. The experiment was repeated twice with similar results.

```
NISO                                    MDSV-SAALVFH----SSIY-LCA     18

PR10-10    MA-H-HGVSGLVGKLVTQLEVNCDADIFYKIVKHHEEVPN-VIPHFFTGVQVTKGDGLVSG     58
NISO       MA-H-HGVSGLVGKIVTELEVNCNADEFYKILKRDEDVPR-AVSDLFPPVKIAKGDGLVSG     76
THS2       MA--PLGVSGLVGKLSTELEVDCDAEKYYNMYKHGEDVKK-AVPHLCVDVKIISGDPTSSG     58
PR10-4     MA-HPHPISGLVGKLVTELEVNCDADKYYKIFKHHEDVPK-AVPHMYTSVKVVEGHGITSG     59
PR10-11    MA-HTRGISGLVGKLVMETEVNCNADKYYQIYKHHEDLPS-AIPHIVTSAKAVEGHGTTSG     59
PR10-12    MARH--GGSGLVGKLVTELEVYCDADKYYKIWKHHEDVPK-AMPHMFTGVQPIKGDGICSG     58
MLP15      MA-HQHTISGLVGKLITESEVNCNADKYYQIFKHHEDLPS-AIPHIYTSVKAVEGHGTTSG     59
MLP1      MMA-H-HGVSGLVGKVVTELELNCDADEYYKVYKHHQLVPNEAVSHLFTGVKALEGGDGLSP     60
MLP2      M------------------------------FKHDENITN-IIPHIYTSFKVVEG-DGLIS     29
MLP3       MAQH-HTISGLIGKLVTESEVNCDAEKYYKIIKHHEDVPN-ATPYVS-DVKVTEGHGTTSG     58
MLP4       MASYDYGLSGLIGKFIIQLEINSDADNFYEIYKHCKDVPK-AVPHLFTGVKVTKGDELVSG     60
PR10-5     MA-H-HGVSGLVGKLVTELEVHCNADAYYKIFKHQEDVPK-AMPHLYTGGKVISGDATRSG     58
PR10-8     MA-H-HGVSGLVGKLVTQLEVNCDADEFYKIWKHHEEVPK-AVSHFFPAVKVVKGDGLVSG     58
PR10-9     MA-H-HGVSGLVGKLVTQLEVNCDADKLYKIYKHHEDVPK-AISHLFTGVKVLEGHGLRSG     58
PR10-14    MA-HHYSTSGLVGKLVTEMEVNCNAENYYQIFKQHEGVPK-AIPHIFTSMKVLEGHGLTSG     59
PR10-15    MA-QPQCISGLSGKLVTKSNVNCGANDFYTIFKQHVDVPK-AIPQIYKCVKVVEGDGTTSG     59

PR10-10   C-IKEWNYVLEGKAMTAVEETTHADETRTLTHHITEGDAMKDYKKFDVIVETNPKPNGHGSV    119
NISO      C-IKEWDCVLDGKAMSGKEETTHNDETRTLRHRELEGDLMKDYKKFDSIIEVNPKPNGHGSI    137
THS2      C-IKEWNVNIDGKTIRSVEETTHDDETKTLRHRVFEGDVMKDFKKFDTIMVVNPKPDGNGCV    119
PR10-4    C-VKEWGYLLEGKELIVKETTTYTDETRTIHHSAVGGHMTKIYKKFDATLVVNPKPSGHGST    120
PR10-11   C-VKEWGYMHEGKTLTCKEKTTYNDETRTICHSISEGDLMNDYKKFDATLVVDPKDNGHGSI    120
PR10-12   S-IKEWNYIIEGKAMRAMEESTHNDETRTISHRVVEGDLLKDYKKFESINEINPKPNGNGCV    119
MLP15     C-VKEWCYILEGKPLTVKEKTTYNDETRTINHNGIEGGMMTDYKKFVATLVVKPKANGQGSI    120
MLP1      VHIKEWSYILEGKTMTAVEESTYDDETRTISHRIVEGDVMKDYKKFDEIVVAKPKPDGHGSI    121
MLP2      GCTKEWGYLSEGKARIVKEQTTFDDETRTIHHCAKAGDMMNDYKKFVLTLVVNPKA--HGQ-     88
MLP3      C-VKQWNFVVAGRNEYVLEKTTYNDETRTICHSDFEGDLMKKYKKFDAILVVKPKDNGHGSN    119
MLP4      C-IKEWNYVLEGKAMTAVEETTIDDATRTLTHHVIEGDVMKDYKKFDVIIEANPKPSGQGTI    121
PR10-5    C-IKEWNYILEGKALIAVEETTHDDETRTLTHRITGGDLTKDYKKFVKIVEVNPKPNGHGSI    119
PR10-8    C-IKEWHYILEGKAMSAMEETTHNDETRTLHHQVVEGEVMKDYKAIASIIQVNPNPNGHGSI    119
PR10-9    C-IKEWKYIIDGKALTAVEETTHGDETRTLKHRVIDGDLMKDYKKFDKIIEANPKPNGHGSI    119
PR10-14   C-IKEWHYLHEGKALKFKETTTYNDEERTICHSVIGGDLLNDYKNFSATLLVKVKPMGHGTT    120
PR10-15   C-IKEWGYHCEGKELIVKEKTTYTDETRTICHCVVGGDIANEYKKFYAILVVNPKPCGN---    117

PR10-14   LAPPVQPAPKQHFSQPAQPASKHHHFSLHRPHLNQPAQPDSKHHLSLHRPHLNLCKTISHCP
                LTGRVLGVQDSSPPAPTYVAPPVPTYVAPPMH                             214

PR10-10   ----VTYSIVYEKINEDSPAPFDYLKFFHQNIVDMSAHI------CSSA----         158
NISO      ----VTWSIEYEKMNEDSPAPFAYLASFHQNVVEVDSHL------CLS--E--         176
THS2      ----VTRSIEYEKTNENSPTPFDYLQFGHQAIEDMNKYL---RDS-----ESN         160
PR10-4    ----VSWTIDYEKINEDSPVPIPYLAFFHKLIEDLNSHL------CA------         158
PR10-11   ----VKYILDYEKINEDSPVPIHYLALCNQATEDLNTYL------CAS--V--         159
PR10-12   ----VTWTIAYEKINEDSPTPFAYIPFVHQAIEDTNKHL------AGS--E--         158
MLP15     ----VTWIVDYEKINEDSPVPFDYLAFFQQNIEDLNSHL------CAS--D--         159
MLP1      ----VSISIMYEKINEDSPTPFDILKTFHQNILDLSAHI------CAS--E--         160
MLP2      -GSTVKWIIDYEKINEDSPVPFAYLSLCIKITEGLNSHI------YAS--E--         130
MLP3      ----VRWTIEYEKNNEDSPVPIDYLGFFQSLIDDLNSHL------CSS-----         158
MLP4      GGSIVTVSIVYDRMNAKSPAPFDYYKFYYQNIVDMDAHI------STS-----         164
PR10-5    ----VTVSLVYEKMNEGSPTPFNYLQFVHQTIVGLNSHI------CAS-----         158
PR10-8    ----VTWSIEYEKMNEDSPTPFAYLEFFHQNIIDMNSHLYVGSDSHLHVDE--         166
PR10-9    ----VTVSLLYEKINEDSPAPFDHLKFFHQNIEDMNSHI------CAS--E--         158
PR10-14   YGSTVMWIIDYEKINKDSPIPVPYLAFFHQIIVDLNSHF-----SASY-----         257
PR10-15   -GSIVSWTVDYEKINKDSPIPIPYIALFARVIEGLDSYL------CAY--A--         159
```

Supplementary Figure 10. Multiple sequence alignment of major latex PR10 and MLP proteins. Residues conserved in PR10-10 are highlighted in black, the binding pocket residues in green, and the conserved disulfide pair in yellow.

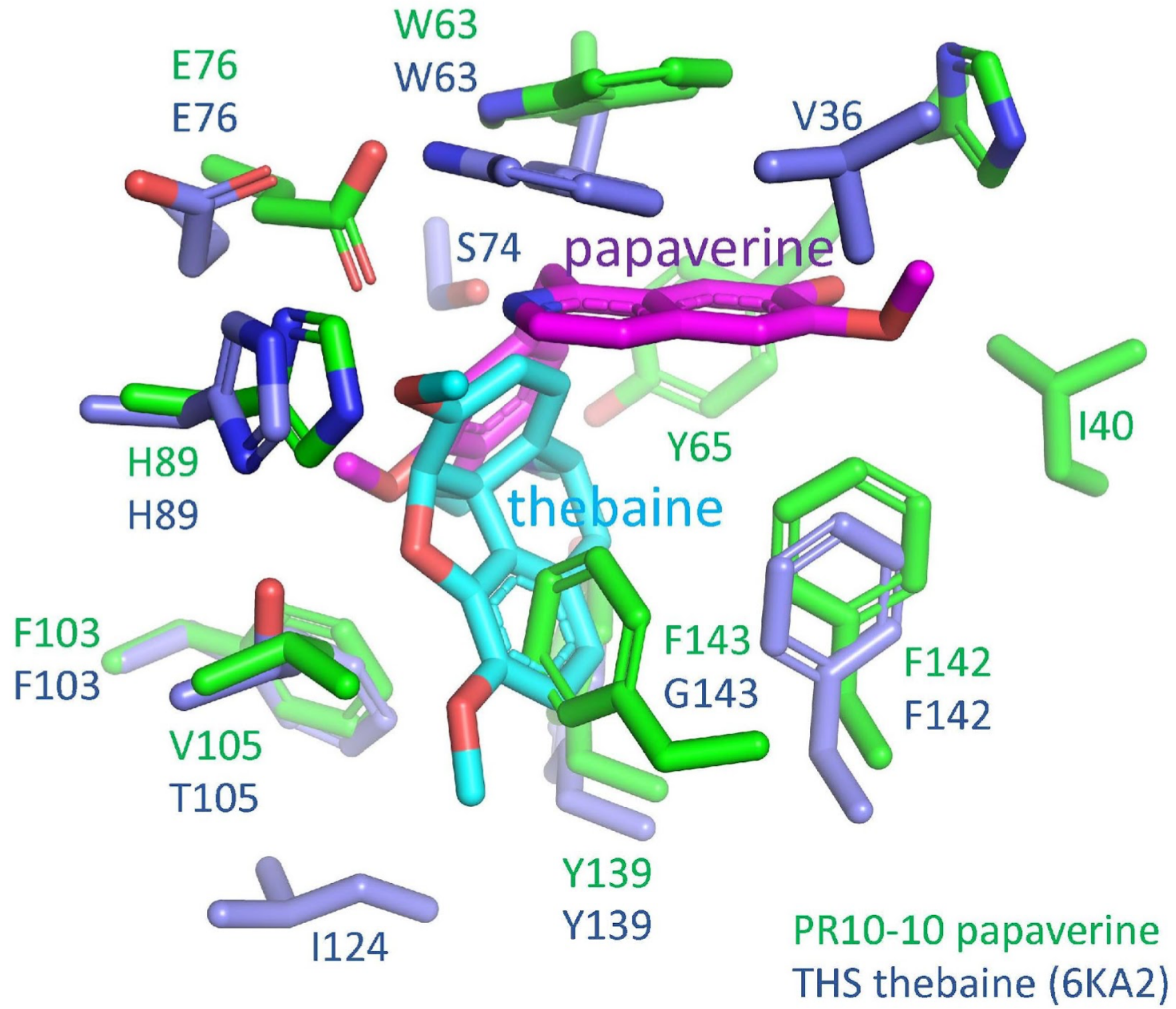

Supplementary Figure 11. Superposition of PR10-10 papaverine complex and THS thebaine complex (6KA2). PR10-10 protein carbons are shown in green and complexed papaverine in magenta. THS protein carbons are shown in blue and complexed thebaine in cyan. Dark blue corresponds to nitrogen atoms, red to oxygen, and yellow to sulfur.

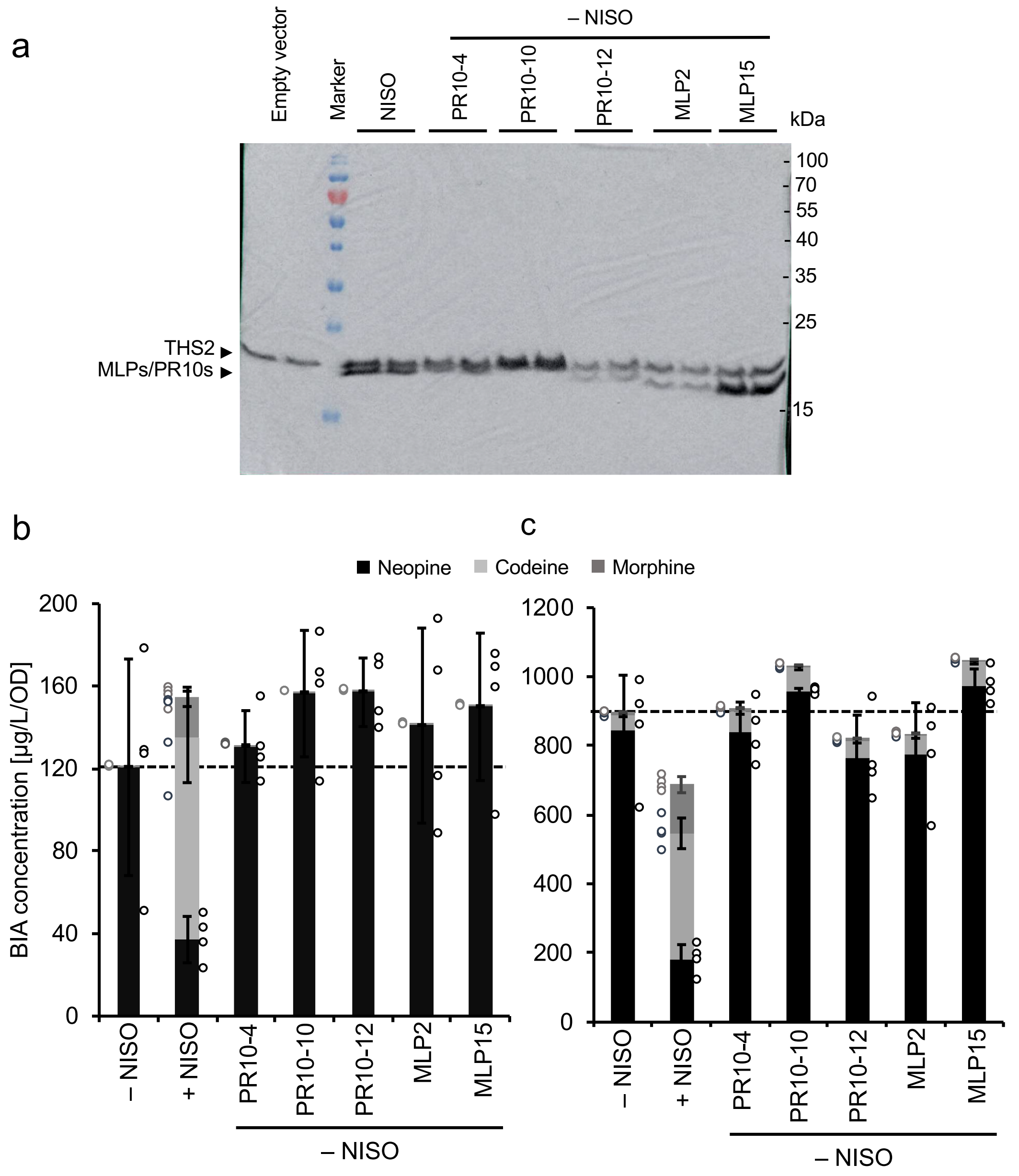

Supplementary Figure 12. Effect of major MLP/PR10 proteins on morphinan alkaloid accumulation in an opiate-producing yeast strain fed thebaine. a, Immunoblot showing the production of recombinant MLP/PR10 proteins in the *NISO* knockout engineered yeast strain. Anti-HA antibodies were used to detect HA-tagged proteins. HA-tagged thebaine synthase (THS) was ubiquitously detected. The experiment was repeated twice with similar results. **b**, Accumulation of morphine, codeine and neopine in engineered yeast fed 50 µM exogenous thebaine for 2h. **c**, Accumulation of morphine, codeine and neopine in engineered yeast fed 50 µM exogenous thebaine for 6h. Bars represent the mean ± standard deviation of 4 biological replicates. Stacked bars show the mean levels of individual morphinan alkaloids ± standard deviation. No significant difference ($p<0.05$; unpaired, two-tailed *t*-test) in alkaloid accumulation was observed.

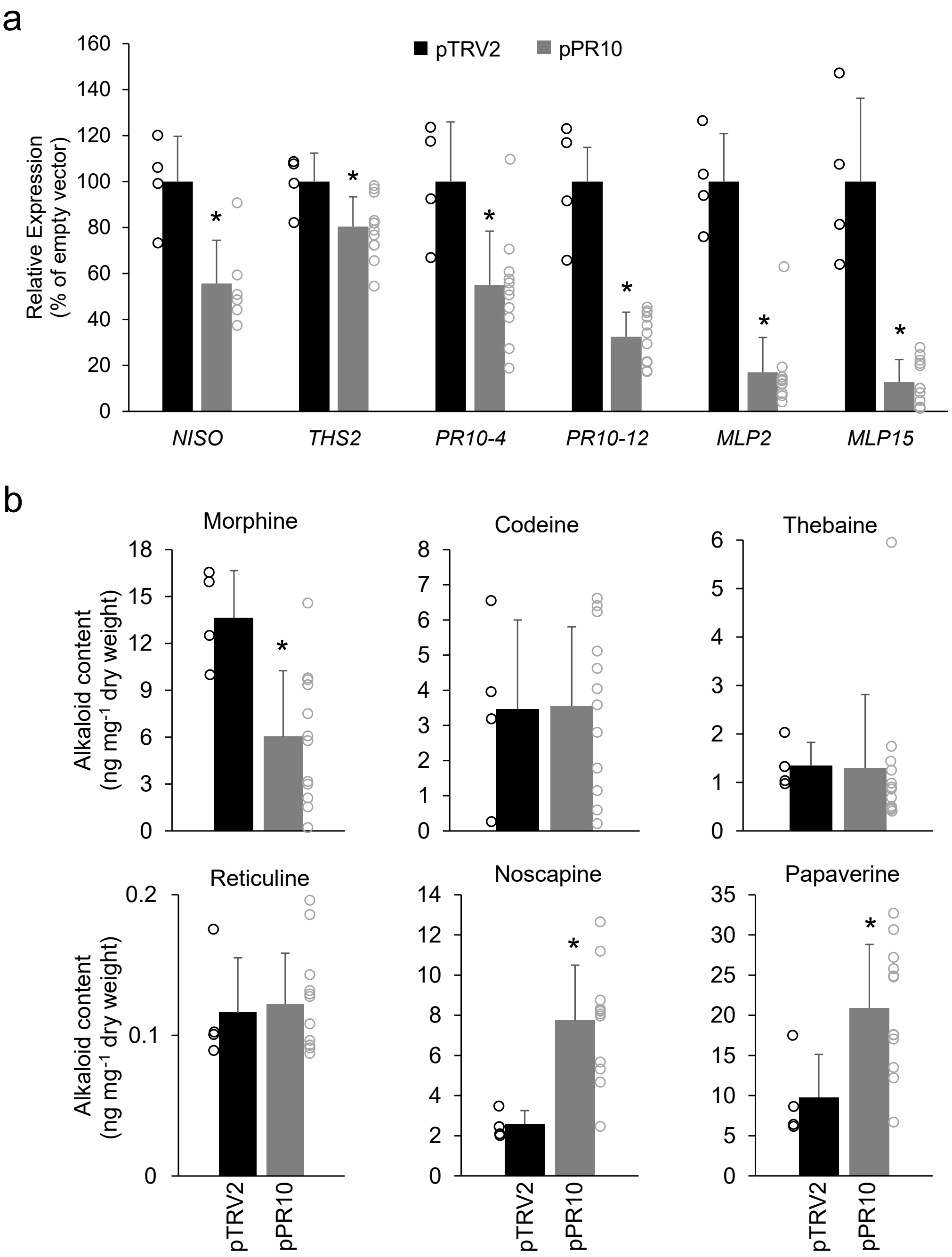

Supplementary Figure 13. Virus-induced gene silencing using the pPR10 construct in the opium poppy Bea's Choice chemotype. a, RT-qPCR analysis of transcripts encoding major MLP/PR10 proteins in control and silenced plants. **b,** Abundance of major alkaloids in control and silenced plants. Bars represent the mean ± standard deviation of 4 and 12 independent biological replicates for control and silenced plants, respectively, and asterisks indicate a significant ($p<0.05$; two-tailed t-test) alkaloid accumulation.

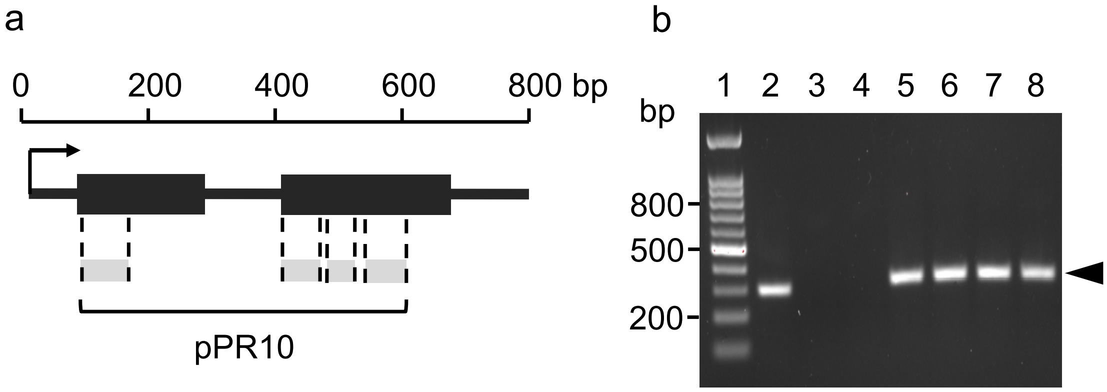

Supplementary Figure 14. Design of pTRV2 construct used for virus-induced gene silencing targeting genes encoding MLP/PR10 proteins and infection screening results. **a**, A generalized diagram of genes encoding MLP/PR10 proteins in opium poppy showing regions used to construct the pTRV2 vector. Black boxes indicate exons and light grey boxes show regions of *MLP15*, *PR10-10,* and *PR10-4* used to build the pPR10 construct. **b**, Representative screen of *Agrobacterium tumefaciens*-infiltrated plants used to identify infected individuals based on the detection of TRV2 coat protein transcripts (arrowhead) by RT-PCR on total RNA extracted from plants 30 days after infiltration. A DNA size marker is shown in lane 1. The experiment was repeated twice with similar results.

Supplementary Table 1. Distribution of organelle markers in sucrose density gradient fractions. Abbreviations: PEPCase, phosphoenolpyruvate carboxylase; PDI, protein disulfide-isomerase; SCS, succinyl-CoA ligase subunit; KAT2, peroxisomal 3-ketoacyl-CoA thiolase 2.

Chemotype 40

		Gradient fraction				
**Marker protein**	**Compartment**	**1**	**2**	**3**	**4**	**5**
PEPCase	Cytosol	n.d.	n.d.	n.d.	n.d.	**0.330%**
PDI	ER	0.170%	0.071%	0.488%	**0.953%**	0.035%
SCS	Mitochondria	0.054%	n.d.	**0.198%**	0.055%	n.d.
KAT2	Peroxisome	n.d.	**0.035%**	0.030%	0.012%	n.d.

n.d. not detected

Chemotype T

		Gradient fraction				
**Marker protein**	**Compartment**	**1**	**2**	**3**	**4**	**5**
PEPCase	Cytosol	0.005%	n.d.	n.d.	n.d.	**0.436%**
PDI	ER	0.300%	0.074%	0.432%	**0.620%**	0.023%
SCS	Mitochondria	0.072%	n.d.	**0.150%**	0.042%	0.003%
KAT2	Peroxisome	n.d.	**0.022%**	0.018%	0.006%	n.d.

n.d. not detected

Chemotype Roxanne

		Gradient fraction				
**Marker protein**	**Compartment**	**1**	**2**	**3**	**4**	**5**
PEPCase	Cytosol	n.d.	n.d.	n.d.	n.d.	**0.409%**
PDI	ER	0.194%	0.193%	0.284%	**0.446%**	0.005%
SCS	Mitochondria	0.048%	0.054%	**0.128%**	0.094%	0.003%
KAT2	Peroxisome	n.d.	**0.065%**	0.018%	0.016%	n.d.

n.d. not detected

Chemotype Przemko

		Gradient fraction				
**Marker protein**	**Compartment**	**1**	**2**	**3**	**4**	**5**
PEPCase	Cytosol	n.d.	n.d.	n.d.	n.d.	**0.374%**
PDI	ER	n.d.	0.015%	0.045%	**0.177%**	0.040%
SCS	Mitochondria	0.066%	0.015%	n.d.	n.d.	n.d.
KAT2	Peroxisome	n.d.	**0.015%**	n.d.	n.d.	n.d.

n.d. not detected

Supplementary Table 2. Relative abundance of MLPs/PR10s in T, 40, Roxanne, Przemko chemotypes.

Chemotype	Total spectra	Total spectrum count of MLPs/PR10s	Relative Abundance
T	100722	3142	0.03290
Roxanne	108064	2954	0.02733
40	95488	2664	0.02645
Przemko	63483	1267	0.01996

Supplementary Table 3. Binding parameters determined from ANS fluorescence and displacement assay curves. Apparent and estimated K_d values were calculated for ANS and the most abundant alkaloids in opium poppy latex, respectively, using seven PR10/MLP proteins. Fitting of a one-site hyperbolic binding model to the ANS data allowed direct calculation of apparent K_d. Protein-ligand combinations for which binding could not be determined, or datasets that produced results displaying substantial error were not determined (nd). Values represent the mean ± standard error of three technical replicates.

Protein	Ligand	K_d (μM)	$Log_{10}IC_{50}$	IC_{50} (μM)	Estimated K_d (μM)	Hill Slope
THS2	ANS	20.85 ± 3.36				
	Thebaine		nd	nd	nd	nd
	Morphine		~ 5.99 ± ~84.77	>1000	nd	-0.66 ± 0.36
	Codeine		nd	nd	nd	
	Papaverine		2.69 ± 0.71	493.17	333.31	-0.88 ± 0.34
	Noscapine		~1.16 ± 0.48	~14.29	~9.66	~-1.13 ± 0.78
NISO	ANS	60.50 ± 7.50				
	Thebaine		2.30 ± 0.14	199.53	171.22	0.92 ± 0.16
	Morphine		1.71 ± 0.05	51.29	44.01	-1.20 ± 0.16
	Codeine		1.45 ± 0.13	28.18	24.19	-0.72 ± 0.13
	Papaverine		1.98 ± 0.05	95.50	81.95	-1.20 ± 0.05
	Noscapine		1.82 ± 0.07	66.07	56.70	-5.05 ± 6.55
PR10-4	ANS	106.70 ± 15.35				
	Thebaine		3.41 ± 1.42	2570.40	2350.14	-0.66 ± 0.26
	Morphine		3.32 ± 0.45	2089.30	1910.26	-1.32 ± 0.76
	Codeine		~7.75 ± ~318	nd	nd	-0.59 ± 0.42
	Papaverine		1.62 ± 0.05	41.69	38.11	-1.32 ± 0.20
	Noscapine		~2.102 ± 0.59	~126.47	~115.64	-0.47 ± 0.17
PR10-10	ANS	nd				
	Thebaine		nd	nd	nd	nd
	Morphine		3.84 ± 2.50	6870.68	nd	-0.31 ± 0.18
	Codeine		~16.45 ± ~2518	nd	nd	~0.13 ± 0.35
	Papaverine		~9.75 ± ~343	nd	nd	-0.36 ± 0.27
	Noscapine		nd	nd	nd	nd
PR10-12	ANS	356.50 ± 139.40				
	Thebaine		2.65 ± 0.21	444.63	432.50	-1.67 ± 0.70
	Morphine		nd	nd	nd	nd
	Codeine		3.18 ± 0.36	1499.68	1458.77	-1.38 ± 0.58
	Papaverine		2.20 ± 0.09	158.49	154.16	-1.70 ± 0.50
	Noscapine		~0.62 ± 0.17	~4.18	~4.07	-0.65 ± 0.16
MLP2	ANS	28.63 ± 3.90				
	Thebaine		2.60 ± 0.20	394.46	292.35	-1.55 ± 0.61
	Morphine		nd	nd	nd	nd
	Codeine		2.31 ± 0.14	203.24	150.62	-0.81 ± 0.16
	Papaverine		1.79 ± 0.07	61.38	45.49	-0.87 ± 0.12
	Noscapine		~1.61 ± 0.07	~40.36	~29.92	~-3.69 ± ~2.82
MLP15	ANS	78.40 ± 10.18				
	Thebaine		2.84 ± 0.23	690.24	612.16	-1.05 ± 0.19
	Morphine		3.08 ± 0.31	1191.24	1056.49	-1.04 ± 0.31
	Codeine		3.02 ± 0.17	1049.54	930.82	-0.97 ± 0.13
	Papaverine		2.48 ± 0.08	299.23	265.38	-1.21 ± 0.08
	Noscapine		1.63 ± 0.04	42.56	37.75	-1.18 ± 0.10

Supplementary Table 4. **Thermodynamic characterization of the interactions between PR10/MLP proteins and various ligands.** Values were determined by isothermal calorimetry (ITC) at pH 5.5 and/or pH 7.4. The heat of dilution was subtracted for each titration. Systems for which no heat of interaction was detected were not determined (nd). An asterisk (*) indicates systems for which there was evidence of interaction, but low enthalpy change (<700 cal/mol) and corresponding poor signal to noise ratio precluded calculation of reliable parameters. A double asterisk (**) indicates systems displaying evidence of interfering exothermic and endothermic interactions that precluded the calculation of reliable parameters. All alkaloids are free bases, except for codeine phosphate and (*S*)-reticuline oxalate. Values represent the mean ± standard error calculated for the model fit to one ITC experimental dataset representative of three replicate experiments showing consistent results.

Protein	Buffer	Ligand	N	K_d (μM)	ΔH (cal/mol)	ΔS (cal/mol/˚K)
THS2	PBS pH 7.4	Thebaine	0.523 ± 0.0156	31.25 ± 1.69	2450 ± 97.84	28.8
		Codeine	0.51 ± 0.0145	26.46 ± 1.85	1868 ± 68.6	27.2
NISO	CBS pH 5.5	Codeine	*	*	*	*
	PBS pH 7.4	Thebaine	0.786 ± 0.0251	18.25 ± 1.89	-2396 ± 110.9	13.6
		Codeine	1.07 ± 0.00547	2.22 ± 0.09	-3556 ± 26.07	13.9
		Morphine	0.83 ± 0.00486	5.46 ± 0.22	-1895 ± 15.67	17.7
		(*S*)-Reticuline	nd	nd	nd	nd
PR10-4	PBS pH 7.4	Codeine	nd	nd	nd	nd
PR10-10	CBS pH 5.5	Papaverine	*	* (225 ± 12)	*	*
		Tetrahydropalmatine	*	* (180 ± 133)	*	*
	PBS pH 7.4	Thebaine	nd	nd	nd	nd
		Codeine	nd	nd	nd	nd
		Morphine	nd	nd	nd	nd
		Papaverine	*	*	*	*
		Tetrahydropapaverine	nd	nd	nd	nd
		Tetrahydropalmatine	*	*	*	*
PR10-12	CBS pH 5.5	Noscapine	*	*	*	*
		Papaverine	*	*	*	*
	PBS pH 7.4	Thebaine	0.54 ± 0.00587	21.55 ± 0.71	5348 ± 82.1	39.3
		Codeine	0.938 ± 0.0615	139.66 ± 13.59	6263 ± 582.6	38.6
MLP15	CBS pH 5.5	Codeine	*	*	*	*
		Noscapine	**	**	**	**
		Papaverine	*	*	*	*
		ANS	1.06 ± 0.0244	55.25 ± 2.8	-14690 ± 497.2	-29.8
	PBS pH 7.4	Thebaine	*	*	*	*
		Codeine	nd	nd	nd	nd

Supplementary Table 5. Crystallographic data collection and refinement statistics for opium poppy PR10-10.

**Data collection statistics**	Apo PR10-10	PR10-10-papaverine	PR10-10-noscapine	PR10-10-(*S*)-tetrahydropapaverine
PDB code	7UQL	7UQM	7UQN	7UQU
Space group	$P2_12_12$	$C222_1$	$C222_1$	$C222_1$
Unit cell dimensions				
a, b, c (Å)	70.18, 92.55, 52.86	48.76, 71.99, 92.47	48.87, 71.56, 91.65	48.51, 71.59, 91.84
α, β, y (º)	90, 90, 90	90, 90, 90	90, 90, 90	90, 90, 90
Wavelength (Å)	0.97946	0.97946	0.97946	0.97946
Resolution (Å)	35.1-1.90 (1.95-1.90)	37.0-1.79 (1.84-1.79)	36.9-1.70 (1.75-1.70)	36.8-1.75 (1.80-1.75)
R_{sym}	0.044 (1.094)	0.030 (0.203)	0.038 (0.418)	0.046 (0.074)
$CC_{1/2}$	1.00 (0.849)	1.00 (0.988)	1.00 (0.969)	0.999 (0.904)
I/σ	27.42 (2.42)	40.73 (9.18)	37.17 (5.34)	22.55 (2.93)
Completeness (%)	98.5 (97.8)	99.0 (97.0)	96.8 (76.9)	99.4 (96.8)
Redundancy	8.7 (8.8)	8.6 (8.1)	12.4 (10.2)	8.6 (8.1)
**Refinement**				
Resolution (Å)	35.1-1.90	37.0-1.79	36.9-1.70	36.8-1.75
Unique reflections	27443	15343	17452	16433
R_{work}/R_{free}	0.2075/0.2474	0.1943/0.2378	0.1921/0.2345	0.1978/0.2310
Total no. of atoms	2349	1289	1308	1239
Protein atoms	2234	1156	1191	1131
Ligand atoms	NA	25	30	25
Water atoms	115	108	87	83
Average *B*-factors (protein)	46.9	32.8	37.2	37.4
Average *B*-factors (Ligand)	NA	42.1	98.0	51.4
Average *B*-factors (water)	48.4	40.3	40.4	41.2
r.m.s.d. from ideal geometry				
Bond length	0.0099	0.0155	0.0133	0.0157
Bond angles	0.95	1.51	1.51	1.48
Ramachandran outliers (%)	0.00	0.00	0.00	0.00
Ramachandran favored (%)	98.52	98.51	97.92	99.28
MolProbity score	1.63	1.83	1.52	1.29
Clashscore	6.16	5.98	6.74	3.51

Supplementary Table 6. Primers used to screen for infected VIGS seedlings and for qPCR analysis.

Primer	Sequence	Purpose
VIGS-CP-F	CTGACTTGATGGACGATTC	TRV coat protein screening
VIGS-CP-R	TGTGTTTGGATTCGCAG	TRV coat protein screening
GAPDH-qF:	CTCATTTGAAGGGTGGAGC	qPCR housekeeper
GAPDH-qR:	GTCATTGCGTGGACAGTGG	qPCR housekeeper
MLP15-qF	AAGCCAAAAGCTAATGGGCAAG	qPCR
MLP15-qR	CAGAGGTGAGAGTTCAAGTCTTCG	qPCR
NISO-qF	CGCGATGAAGATGTTCCACG	qPCR
NISO-qR	CTTCCAATTCACGGTGACGC	qPCR
THS2-qF	CGGATGGAAATGGATGTGTT	qPCR
THS2-qR	AACACCGAATGGGTTCATGT	qPCR
PR10-4-qF	GGAGGACACGTGACGAAGAT	qPCR
PR10-4-qR	CCAACAGCTGAAATTGTCCA	qPCR
PR10-12-qF	ATGCGTCGTGACATGGACTA	qPCR
PR10-12-qR	CGTGCATGAGCATACGTACC	qPCR
MLP2-qF	TGGACAAGGAAGCACAGTCAAG	qPCR
MLP2-qR	ACCTATTCGGAAGCGTAGATGTG	qPCR
