# Supplementary material for: Alkaloid binding to opium poppy major latex proteins triggers structural modification and functional aggregation
Source: Nat Commun. 2022 Nov 9;13:6768. doi: 10.1038/s41467-022-34313-6 (PMC9646721; doi:10.1038/s41467-022-34313-6)
Supplement: Supplementary file 2 — Description of Additional Supplementary Files [file 41467_2022_34313_MOESM2_ESM.pdf]

Description of Additional Supplementary Files

Supplementary Data 1: Composition and relative abundance of specific proteins in gradient fractions were determined using comparative shotgun proteomics.

Supplementary Data 2: Nucleotide sequences of the pTRV2-GFP vector and the pPR10 insert used for virus-induced gene silencing experiments.
