# Supplementary material for: Alkaloid binding to opium poppy major latex proteins triggers structural modification and functional aggregation
Source: Nat Commun. 2022 Nov 9;13:6768. doi: 10.1038/s41467-022-34313-6 (PMC9646721; doi:10.1038/s41467-022-34313-6)
Supplement: Supplementary file 5 — Reporting Summary [file 41467_2022_34313_MOESM5_ESM.pdf]

nature portfolio

Corresponding author(s): Peter Facchini
Last updated by author(s): Oct 19, 2022

Reporting Summary

Nature Portfolio wishes to improve the reproducibility of the work that we publish. This form provides structure for consistency and transparency in reporting. For further information on Nature Portfolio policies, see our Editorial Policies and the Editorial Policy Checklist.

Statistics

For all statistical analyses, confirm that the following items are present in the figure legend, table legend, main text, or Methods section.

n/a	Confirmed	
[ ]	[x]	The exact sample size (*n*) for each experimental group/condition, given as a discrete number and unit of measurement
[ ]	[x]	A statement on whether measurements were taken from distinct samples or whether the same sample was measured repeatedly
[ ]	[x]	The statistical test(s) used AND whether they are one- or two-sided<br>*Only common tests should be described solely by name; describe more complex techniques in the Methods section.*
[x]	[ ]	A description of all covariates tested
[x]	[ ]	A description of any assumptions or corrections, such as tests of normality and adjustment for multiple comparisons
[ ]	[x]	A full description of the statistical parameters including central tendency (e.g. means) or other basic estimates (e.g. regression coefficient) AND variation (e.g. standard deviation) or associated estimates of uncertainty (e.g. confidence intervals)
[ ]	[x]	For null hypothesis testing, the test statistic (e.g. *F*, *t*, *r*) with confidence intervals, effect sizes, degrees of freedom and *P* value noted<br>*Give P values as exact values whenever suitable.*
[x]	[ ]	For Bayesian analysis, information on the choice of priors and Markov chain Monte Carlo settings
[x]	[ ]	For hierarchical and complex designs, identification of the appropriate level for tests and full reporting of outcomes
[x]	[ ]	Estimates of effect sizes (e.g. Cohen's *d*, Pearson's *r*), indicating how they were calculated

Our web collection on statistics for biologists contains articles on many of the points above.

Software and code

Policy information about availability of computer code

Data collection	n/a
Data analysis	Transcriptome assembly: Trinity Assembler35 and associated RSEM (version 1.3.1), and R/Bioconductor (version 3.8)<br>Proteomics: RawConverter (version 1.1.0.18; Scripps Research Institute), Mascot (version 2.4; Matrix Sciences), and Scaffold (version 5, Proteome Software Inc)<br>Metabolite LC-MS/MS analysis: Agilent MassHunter Qualitative Analysis Software (version B.06.01)<br>ANS displacement assays: curve fitting and IC50 calculation, GraphPad Prism 5<br>ITC: binding isotherms; MicroCal Origin 7 (MicroCal)<br>X-ray crystallography: Auto XDS, PHASER, PHENIX, COOT, Molprobity, eLBOW, proDRG<br>Statistical analysis: yeast assays, Microsoft Excel; VIGS gene expression and metabolite data, GraphPad Prism 5

For manuscripts utilizing custom algorithms or software that are central to the research but not yet described in published literature, software must be made available to editors and reviewers. We strongly encourage code deposition in a community repository (e.g. GitHub). See the Nature Portfolio guidelines for submitting code & software for further information.

Data

Policy information about availability of data

All manuscripts must include a data availability statement. This statement should provide the following information, where applicable:

- Accession codes, unique identifiers, or web links for publicly available datasets
- A description of any restrictions on data availability
- For clinical datasets or third party data, please ensure that the statement adheres to our policy

The proteomics datasets have been deposited in PRIDE repository (https://www.ebi.ac.uk/pride/) under the dataset identifier (PXD035860). The structures determined by X-ray crystallography are available in the Protein Data Bank (https://www.rcsb.org) under the accession codes (7UQL, 7UQM, 7UQN, and 7UQO). Source data are provided with this paper.

Human research participants

Policy information about studies involving human research participants and Sex and Gender in Research.

Reporting on sex and gender	This study did not involve human research participants
Population characteristics	This study did not involve human research participants
Recruitment	This study did not involve human research participants
Ethics oversight	This study did not involve human research participants

Note that full information on the approval of the study protocol must also be provided in the manuscript.

Field-specific reporting

Please select the one below that is the best fit for your research. If you are not sure, read the appropriate sections before making your selection.

☒ Life sciences ☐ Behavioural & social sciences ☐ Ecological, evolutionary & environmental sciences

For a reference copy of the document with all sections, see nature.com/documents/nr-reporting-summary-flat.pdf

Life sciences study design

All studies must disclose on these points even when the disclosure is negative.

Sample size	The selection of sample sizes was based on our extensive experience with respect to the variation inherent to each of the experimental systems described in our manuscript. For in vivo yeast engineering experiments, four fully independent replicates are standard and sufficient. For in planta gene-silencing experiments 4 and 12 different plants were used for the control and experimental populations, respectively.
Data exclusions	*Describe any data exclusions. If no data were excluded from the analyses, state so OR if data were excluded, describe the exclusions and the rationale behind them, indicating whether exclusion criteria were pre-established.*
Replication	All experiments could be reliably replicated.
Randomization	Plants were selected randomly for gene silencing and proteomics experiments.
Blinding	Group allocation was not relevant to most experiments.

Reporting for specific materials, systems and methods

We require information from authors about some types of materials, experimental systems and methods used in many studies. Here, indicate whether each material, system or method listed is relevant to your study. If you are not sure if a list item applies to your research, read the appropriate section before selecting a response.

Materials & experimental systems

n/a	Involved in the study	
☐	☒	Antibodies
☒	☐	Eukaryotic cell lines
☒	☐	Palaeontology and archaeology
☒	☐	Animals and other organisms
☒	☐	Clinical data
☒	☐	Dual use research of concern

Methods

n/a	Involved in the study	
☒	☐	ChIP-seq
☒	☐	Flow cytometry
☒	☐	MRI-based neuroimaging

Antibodies

Antibodies used	HA-tag (GenScript; cat# A01244) primary antibody and goat anti-mouse (BioRad; cat# 170-5047) secondary antibodies were used at dilutions of 1:1000 and 1:20000, respectively.
Validation	Antibody validation is available from the suppliers.
